# Risk of Stroke Among Different Metabolic Obesity Phenotypes: A Systematic Review and Meta-Analysis

**DOI:** 10.3389/fcvm.2022.844550

**Published:** 2022-04-25

**Authors:** Miaomiao Meng, Yixin Guo, Zhuoran Kuang, Lingling Liu, Yefeng Cai, Xiaojia Ni

**Affiliations:** ^1^The Second Clinical School, Guangzhou University of Chinese Medicine, Guangzhou, China; ^2^Guangdong Provincial Hospital of Chinese Medicine, Guangdong Provincial Academy of Chinese Medical Sciences, Guangzhou, China

**Keywords:** obesity, stroke, risk factor, meta-analysis, metabolic health

## Abstract

**Background and Purpose:**

Overweight/obesity is a modified risk factor for stroke. This systematic review and meta-analysis aimed to assess the impact of different obesity phenotypes on stroke risk in adults.

**Methods:**

The PubMed, Embase, and Cochrane Library databases were searched from their inception to 7 March 2021 to identify the prospective cohort studies investigating stroke risk among different metabolic overweight/obesity phenotypes. The methodological quality of the included studies was evaluated using the Newcastle–Ottawa Scale. Pooled hazard ratios (HRs) with 95% confidence intervals (CIs) were calculated using a random-effects model.

**Results:**

A total of eleven prospective cohorts (*n* = 5,609,945 participants) were included in the systematic review, nine of which were included in the meta-analysis. All metabolically unhealthy phenotypes had a higher risk of stroke than the metabolically healthy normal-weight phenotypes, including metabolically unhealthy normal weight (HR = 1.63, 95% CI: 1.41–1.89, *I^2^* = 89.74%, *n* = 7 cohort studies, 1,042,542 participants), metabolically unhealthy overweight (HR = 1.94, 95% CI: 1.58–2.40, *I^2^* = 91.17%, *n* = 4 cohort studies, 676,166 participants), and metabolically unhealthy obese (HR = 1.99, 95% CI: 1.66–2.40, *I^2^* = 93.49%, *n* = 6 cohort studies, 1,035,420 participants) phenotypes. However, no risk of stroke was observed in the populations with metabolically healthy overweight (MHOW) (HR = 1.07, 95% CI: 1.00–1.14, *I^2^* = 69.50%, *n* = 5 studies, 4,171,943 participants) and metabolically healthy obese (MHO) (HR = 1.07, 95% CI: 0.99–1.16, *I^2^* = 54.82%, *n* = 8 studies, 5,333,485 participants) phenotypes. The subgroup analyses for the MHO studies suggested that the risk of stroke increased only when the MHO participants were mainly females, from North America, and when the World Health Organization standard was applied to define obesity. In the subgroup analysis of the risk of stroke in MHOW, a longer follow-up duration was also associated with a higher risk of stroke.

**Conclusion:**

The risk of stroke increase for all metabolically unhealthy phenotypes irrespective of the body mass index (BMI). The associated risk of stroke with metabolic health but high BMI shows substantial heterogeneity, which requires future research considering the impact of sex and transition of the metabolic status on the risk of stroke.

**Systematic Review Registration:**

The study protocol was prospectively registered in PROSPERO (No. CRD42021251021).

## Introduction

The Global Burden of Diseases, Injuries, and Risk Factors Study (GBD) 2019 showed that stroke is the second leading cause of death globally, and the incidence and prevalence of stroke have grown substantially (1). The identification and control of risk factors could be an effective approach for preventing new stroke cases. Global evidence has shown that obesity is a modifiable risk factor for all types of stroke (2, 3). The GBD 2019 study revealed that obesity is the fastest-growing risk factor in high body mass index (BMI) (1). These findings have been included in clinical practice guidelines in either the West or the East, and weight reduction in overweight/obese individuals is recommended to reduce the risk of stroke (4, 5). However, obesity often coexists with other cardiometabolic risk factors such as hypertension, hyperglycemia, and dyslipidemia (6–9). A meta-analysis of 97 prospective cohorts found that the partial excess risk of overweight and obesity for stroke was mediated by metabolic factors, including high blood pressure, cholesterol, and glucose (10). Thus, co-controlling metabolic factors are gaining attention to achieve optimal body weight, compared with the previous goal of an appropriate BMI only (6–9).

In the last 15 years, a concept that classifies individuals into six phenotypes based on the BMI and metabolic health has been gradually developed and supported by animal and human studies: metabolically healthy normal weight (MHNW), metabolically healthy overweight (MHOW), metabolically healthy obese (MHO), metabolically unhealthy normal weight (MUNW), metabolically unhealthy overweight (MUOW), and metabolically unhealthy obese (MUO) (11).

Whether metabolically healthy overweight/obese increases the cardio-cerebrovascular risk is a substantial debate (12–15). A recent meta-analysis of eight prospective cohorts revealed that MHO is positively associated with an elevated risk of stroke (16). However, it only calculated the risk ratio at the end of the follow-up, which might have omitted the time-dependent effect. It did not perform a subgroup analysis considering the demographic factors and the coexisting health conditions of the participants. Recently, more prospective cohorts of the same interest have been published (17–19). We updated the literature search and conducted a meta-analysis to examine the causal relationship between the obesity phenotypes and stroke risk.

## Materials and Methods

The systematic review and meta-analysis were conducted according to the guidelines of the Preferred Reporting Items for Systematic Reviews and Meta-Analyses (PRISMA) statement (20). The study protocol was prospectively registered in PROSPERO (No. CRD42021251021).

### Eligibility

Studies were included in the systematic review if they met the following criteria:

∎ Type of study

Only the prospective cohort study was eligible as it is the most appropriate design for examining the causal relationship between exposure and risk of disease. A pilot search found that a considerable number of prospective cohorts were published.

∎ Participants

The participants at baseline were adults (≥18 years old) without a preexisting stroke.

∎ Exposure and control

The exposure group had one of the following phenotypes: MHOW, MHO, MUNW, MUOW, or MUO, and the reference group was MHNW. Overweight/obesity was defined by BMI in compliance with the standards from one of the following organizations: the International Obesity Task Force (IOTF), Working Group on Obesity in China (WGOC), and the World Health Organization (WHO). Currently, there is no consensus regarding the definition of metabolic health. We endorsed the methodology of the previous systematic reviews (16), and *”metabolic health was defined as individuals with less than two abnormal metabolic parameters.”* This enabled a direct comparison between the previous evidence and our study.

∎ Outcomes

The primary outcome was the first-ever stroke, which the participants could report—a clinical diagnosis confirmed by clinical signs/symptoms accompanied by brain images or a diagnosis in the medical records/national databases. The patients with ischemia, hemorrhage, or mixed stroke were eligible.

Studies were excluded if they were repeatedly published.

### Search Strategy and Study Selection

We searched the PubMed, Embase, and the Cochrane Library databases from their inception to 7 March 2021. The search strategy is presented in Supplementary Table 1. Two researchers (MMM and GYX) independently screened the literature by removing duplicates, reading the title/abstract, and reviewing the full text. We calculated the inter-rater agreement with the reference of kappa value, and the disagreements were resolved through discussion or consultation with a third researcher (NXJ).

### Data Extraction

Two researchers (MMM and GYX) extracted the data using the double-entry method and were checked by a third researcher (NXJ). The data items included author, publication year, country, sample size, mean or range of age, sex, period of baseline data collection, follow-up duration, overweight/obesity definition, definition of (un)metabolic health, the confounding factors that were adjusted for in the multivariable analysis, and adjusted or non-adjusted effect sizes [hazard ratio (HR) and relative risk (RR)] with the corresponding 95% confidence intervals (CIs).

### Methodological Quality Assessment

Two researchers (KZR, LLL) independently evaluated the methodological quality of the included studies using the Newcastle–Ottawa Scale (NOS), a tool for assessing the quality of observational studies in meta-analyses (21). It was composed of three domains (eight items): selection of the study groups, comparability, and ascertainment of the outcomes for the cohort study. The assessment outcomes ranged from zero stars up to nine stars. Studies with seven or more stars were considered high quality. We calculated the inter-rater agreement concerning the intraclass correlation coefficient (ICC), and any disagreement was resolved by discussion or consultation with a third researcher (MMM).

### Statistical Analysis

Pooled HRs with 95% CIs were calculated using a random-effects model to assess the risk of stroke for MHOW, MHO, MUNW, MUOW, and MUO compared to MHNW. The heterogeneity was evaluated using Cochran’s *Q* test and *I*^2^ statistics. If the heterogeneity was considered significant (*p* < 0.1 or *I^2^* > 50%), subgroup analyses were performed according to the duration of the follow-up, number of abnormal metabolic components, criteria for defining overweight/obesity, research sites, and proportion of females to explore the potential sources of heterogeneity. A sensitivity analysis was performed based on the methodological quality of the included studies and the leave-one-out method. The publication bias was evaluated by the visual inspection of asymmetry in the funnel plots and Egger’s test for at least 10 studies. The statistical significance was set at *p* < 0.05. All data analyses were conducted using Stata/MP version 16.0.

## Results

### Search Results

**FIGURE 1:**
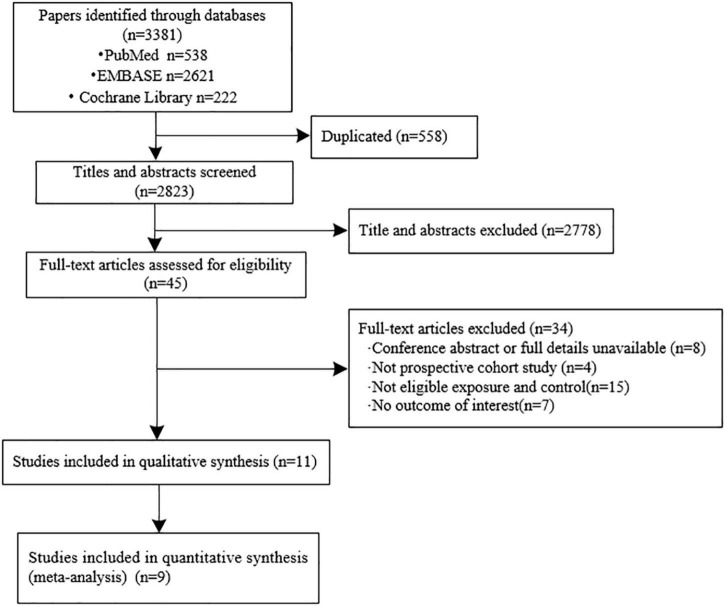
Flow chart for the literature search, study selection, and reasons for exclusion.

We initially identified 3,381 records from the database search and finally included 11 eligible cohort studies in the systematic review (13–15, 17–19, 22–26). The inter-examiner agreement between the two reviewers was good (kappa = 0.762, 95% CI: 0.5464–0.9776). The results from nine cohorts were quantitatively synthesized as two studies reported the relative ratio (RR) instead of HR (22, 25). A full flowchart of the study selection process is shown in Figure 1. A list of the excluded studies is shown in Supplementary Data Sheet 1.

### Study Characteristics

The sample sizes of the included cohorts ranged from 5,171 to 3,495,777 participants. The included studies were conducted in different geographic locations: four studies were conducted in Europe (14, 15, 24, 25), two in North America (13, 26), and five in Asia (17–19, 22, 23). Female participants were enrolled in only three studies (13, 25, 26). The baseline information was collected between 1980 and 2018, and the duration of the follow-up ranged from 3 to 24 years. The BMI threshold for obesity varied, including BMI ≥ 25 kg/m^2^ (International Obesity Task Force, IOTF) in two studies (17, 23), BMI ≥ 28 kg/m^2^ (Working Group on Obesity in China, WGOC) in three studies (18, 19, 22), and BMI ≥ 30 kg/m^2^ (World Health Organization, WHO) in six studies (13–15, 24–26). The definition of metabolic health was distinct, such as the Adult Treatment Panel III (ATP III) definition in three studies (14, 15, 26), the International Diabetes Federation (IDF) in one study (18), the Japanese diagnostic criteria in one study (17), modified harmonization and the Hammer’s study in three studies (19, 22, 23). The stroke event was reported by the participants in another study (22), diagnosed according to the clinical signs/symptoms and brain images in four studies (13, 14, 23, 24), and was collected from the medical records and registration information in the national databases. The full details of the basic characteristics of the included cohorts are presented in Table 1.

**TABLE 1:** Characteristics of the included studies.

Study	Country/Panel	Funding	Sample size	Age	Female (%)	Baseline collection period	Follow up	Definition of obesity	Definition of metabolic health	Adjusted variables in analyses
Hidetaka (17)	Japan/The Japan Medical Data Center (JMDC)	the Ministry of Health, Labour and Welfare, Japan and the Ministry of Education, Culture, Sports, Science and Technology, Japan	802288	42.8 ± 9.4	55.3%	2005∼2018	1126 ± 849 days	BMI ≥ 25 kg/m^2^ from the criteria by IOTF	Metabolic health was defined as none of the risk factors according to the Japanese diagnostic criteria.	Unadjusted
Zhou (18)	China/The Zhejiang Metabolic Syndrome cohort and the Kailuan cohort	the National Key Research and Development Program of China and Hangzhou Science and Technology Project	102037	51.5 ± 12.8	26.7%	Zhejiang: 2010∼2014 Kailuan: 2006∼2007	9.9 ± 2.0 years	BMI ≥ 28 kg/m^2^ from the criteria by WGOC	Metabolic health was defined as ≤1 of the risk factors according to IDF criteria.	age, sex, smoking, drinking, physical activity, family history of stroke
Gao (19)	China/The China Kadoorie Biobank cohort	the National Key R&D Program of China, the United Kingdom Wellcome Trust, National Natural Science Foundation of China and Chinese Ministry of Science and Technology	458246	50.9 ± 10.4	59.2%	2004∼2008	10.0 years	BMI ≥ 28 kg/m^2^ from the criteria by WGOC	Metabolic health was defined as ≤1 of the risk factors according to modified harmonization definition.	age, region, sex, education, household income, marital status, smoking, drinking, intakes of red meat, fresh fruits, and vegetables, physical activity, family history of heart attack or stroke
Li (22)	China/The China Health and Retirement Longitudinal Study (CHRLS)	the National Institute on Aging, the National Natural Science Foundation of China, the World Bank and Peking University	7849	59.88 ± 10.82	52.8%	2011∼2012	3.67 years	BMI ≥ 24 kg/m^2^ (overweight/obesity) from the criteria by WGOC	Metabolic health was defined as ≤1 of the risk factors according to Hammer’s study.	age, gender, residence, educational level, marital status, smoking, drinking, physical activity, history of arthritis, asthma and history of fall, physical impairments in ADL and IADL, and cognition score, total cholesterol, high-density cholesterol levels
Lee (23)	Korea/The Korean National Health Insurance Service–National Sample Cohort (NHIS-NSC)	the Seoul National University Hospital Research Fund, the National Research Foundation of Korea at Ministry of Education, Science and Technology and the Korean Healthcare Technology R&D project at the Ministry of Health and Welfare	354083	45.8 ± 14.2	47.3%	2004∼2008	7.43 ± 1.52 years	BMI ≥ 25 kg/m^2^ from the criteria by IOTF	Metabolic health was defined as none of the risk factors according to modified harmonization definition.	age, sex, income, area, smoking, drinking, exercise, history of ischemic heart disease, peripheral artery disease, congestive heart failure, transient ischemic attack, venous thromboembolism, chronic obstructive pulmonary disease, end-stage renal disease, liver cirrhosis, cancer, and cardiac surgery
Nathalie (13)	United States/ The Nurses’ Health Study (NHS)	the United States National Institutes of Health and the German Federal Ministry of Education and Research (BMBF)	90257	NA	100%	1980	24 years	BMI ≥ 30 kg/m^2^ from the criteria by WHO	Metabolic health was defined as none of the metabolic disorders (hypertension, diabetes, and hypercholesterolemia).	age, race, highest educational degree, alcohol consumption, smoking status, post-menopausal status, post-menopausal hormone use, physical examinations for screening purposes, aspirin use, family history of myocardial infarction and diabetes and physical activity
Caleyachetty (24)	United Kingdom/ The Health Improvement Network (THIN)	No mention	3495777	NA	54.5%	1995∼2015	5.4 years	BMI ≥ 30 kg/m^2^ from the criteria by WHO	Metabolic health was defined as none of the metabolic disorders (hypertension, diabetes, and hypercholesterolemia).	age, sex, smoking status and social deprivation
Laura (14)	Spain/The Vascular-Metabolic CUN cohort	No financial support	5171	men: 55.61 ± 13.68 women: 54.21 ± 12.87	38.1%	1997∼2002	Men: 9.18 years women: 8.97 years	BMI ≥ 30 kg/m^2^ from the criteria by WHO	Metabolic health was defined as ≤1 of the risk factors according to ATP III criteria.	age, sex, BMI, cigarette smoking, daily alcohol intake, lifestyle pattern, hypertension, coronary heart disease, type 2 diabetes, anti-aggregation therapy, HDL-cholesterol, LDL-cholesterol, and triglycerides
Hinnouho (15)	United Kingdom/The Whitehall II study	the United States National Institutes of Health, the United Kingdom Medical Research Council, the Economic and Social Research Council and the British Heart Foundation	7122	49.3	30.3%	1991∼1993	17.4 years	BMI ≥ 30 kg/m^2^ from the criteria by WHO	Metabolic health was defined as ≤1 of the risk factors according to ATP III criteria.	sex, socioeconomic status, marital status, ethnicity, physical activity, smoking, alcohol, fruits and vegetables consumption, CVD medication and procedures
Andersen (25)	Denmark/Danish Medical Birth Register	the University of Copenhagen, Denmark, the Danish Agency of Science, Technology and Innovation and the Novo Nordisk Foundation	261489	30.5	100%	2004∼2009	5.6 years	BMI ≥ 30 kg/m^2^ from the criteria by WHO	Metabolic health was defined as none of the risk factors (any hypertensive disorder, any abnormality in glucose metabolism and dyslipidaemia).	age, calendar year and smoking
Song (26)	United States/The Women’s Health Study	the National Institutes of Health, Bethesda, Maryland and an American Diabetes Association Career Development Award, Alexandria, Virginia	25626	NA	100%	1992∼1995	10.2 years	BMI ≥ 30 kg/m^2^ from the criteria by WHO	Metabolic health was defined as ≤2 of the risk factors according to ATP III criteria.	smoking, exercise, alcohol intake, total calorie intake, postmenopausal hormone use, multivitamin use and parental history of myocardial infarction at age <60 years

*IOTF, International Obesity Task Force; WGOC, Working Group on Obesity in China; WHO, World Health Organization; ATP III, Adult Treatment Panel III; IDF, International Diabetes Federation; NA, not reported.*

### Methodological Quality Assessment

The inter-examiner agreement was very good (ICC = 0.771, 95% CI: 0.3503–0.933). Regarding the methodological quality of the included studies, one study was awarded six stars (17), three studies evaluated eight stars (13, 15, 22), and seven studies achieved nine stars (14, 18, 19, 23–26). The average quality score was eight, suggesting that the methodological quality was generally moderate to high. The results of the quality assessments are presented in Table 2.

**TABLE 2:** Quality evaluation of the included studies.

Study	Selection of the study groups				Comparability of the groups	Ascertainment of outcome			Sum	Overall quality
	1	2	3	4	5	6	7	8		
Hidetaka (17)	**√**	**√**	**√**	**√**		**√**		**√**	6	Moderate
Zhou (18)	**√**	**√**	**√**	**√**	**√√**	**√**	**√**	**√**	9	High
Gao (19)	**√**	**√**	**√**	**√**	**√√**	**√**	**√**	**√**	9	High
Li (22)	**√**	**√**	**√**	**√**	**√√**	**√**		**√**	8	High
Lee (23)	**√**	**√**	**√**	**√**	**√√**	**√**	**√**	**√**	9	High
Nathalie (13)		**√**	**√**	**√**	**√√**	**√**	**√**	**√**	8	High
Caleyachetty (24)	**√**	**√**	**√**	**√**	**√√**	**√**	**√**	**√**	9	High
Laura (14)	**√**	**√**	**√**	**√**	**√√**	**√**	**√**	**√**	9	High
Hinnouho (15)		**√**	**√**	**√**	**√√**	**√**	**√**	**√**	8	High
Andersen (25)	**√**	**√**	**√**	**√**	**√√**	**√**	**√**	**√**	9	High
Song (26)	**√**	**√**	**√**	**√**	**√√**	**√**	**√**	**√**	9	High

*1: Representativeness of the exposed cohort; 2: Selection of the non-exposed cohort; 3: Ascertainment of exposure; 4: Demonstration that outcome of interest was not present at start of study; 5: Comparability of cohorts on the basis of the design or analysis; 6: Assessment of outcome; 7: Was follow-up long enough for outcomes to occur; 8: Adequacy of follow up of cohorts.*

### Results of the Meta-Analyses

#### Metabolically Healthy Overweight/Metabolically Healthy Obese and Risk of Stroke

**FIGURE 2:**
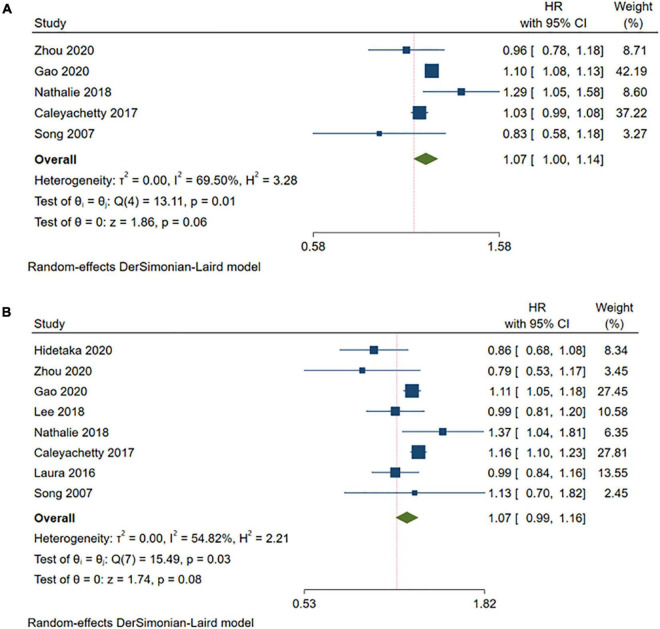
Meta-analysis of the risk of stroke in the MHOW/MHO phenotypes compared with the MHNW phenotypes. **(A)** MHOW phenotypes; **(B)** MHO phenotypes. MHNW, metabolically healthy normal weight; MHOW, metabolically healthy overweight; MHO, metabolically healthy obese; MUNW, metabolically unhealthy normal weight; MUOW, metabolically unhealthy overweight; MUO, metabolically unhealthy obese.

Compared with the MHNW group, the risk of stroke was not significantly increased in the participants with MHOW (MHOW: HR = 1.07, 95% CI: 1.00–1.14, *I^2^* = 69.50%, *n* = 5 studies, 4,171,943 participants) (13, 18, 19, 24, 26) or MHO (HR = 1.07, 95% CI: 0.99–1.16, *I^2^* = 54.82%, *n* = 8 studies, 5,333,485 participants) (13, 14, 17–19, 23, 24, 26) (Figure 2 and Supplementary Table 2). The subgroup analyses for the risk of stroke in the MHO were conducted in terms of the duration of the follow-up, the number of abnormal metabolic parameters, the definition of obesity, geographic locations, and proportion of females (Table 3 and Supplementary Figures 1–5), which revealed that the risk of stroke increased only when the MHO participants were mainly females, from North America, and when the WHO standard was applied in defining obesity, and these meta-analyses were of low heterogeneity. As for the subgroup analysis of the risk of stroke in the MHOW, a longer follow-up duration was also associated with a higher risk of stroke (Table 3 and Supplementary Figures 6–10).

**TABLE 3:** Subgroup analysis for the association between the metabolic phenotypes and risk of stroke.

Potential moderators	Subgroup	Number of studies	Number of participants	HR (95% CI)	*I*^2^ (%)
**MHOW**					
Follow up	<9 years	1 (24)	3495777	1.03 (0.99–1.08)	/
	9∼15 years	3 (18, 19, 26)	585909	1.02 (0.89–1.18)	51.07
	>15 years	1 (13)	90257	1.29 (1.05–1.58)	/
Number of risk factors in metabolic health	0	2 (13, 24)	3586034	1.13 (0.91–1.40)	77.56
	≤1	2 (18, 19)	560283	1.07 (0.96–1.19)	41.53
	≤2	1 (26)	25626	0.83 (0.58–1.18)	/
Criteria for defining obesity	IOTF	0	0	/	/
	WGOC	2 (18, 19)	560283	1.07 (0.96–1.19)	41.53
	WHO	3 (13, 24, 26)	3611660	1.06 (0.89–1.28)	66.6
Region	Asia	2 (18, 19)	560283	1.07 (0.96–1.19)	41.53
	North America	2 (13, 26)	115883	1.06 (0.69–1.63)	77.53
	Europe	1 (24)	3495777	1.03 (0.99–1.08)	/
Proportion of female	<40%	1 (18)	102037	0.96 (0.78–1.18)	/
	40%∼90%	2 (19, 24)	3954023	1.07 (1.00–1.14)	85.24
	100%	2 (13, 26)	115883	1.06 (0.69–1.63)	77.53
**MHO**					
Follow up	<9 years	3 (17, 23, 24)	4652148	1.02 (0.85–1.23)	75.37
	9∼15 years	4 (14, 18, 19, 26)	591080	1.05 (0.95–1.17)	30.21
	>15 years	1 (13)	90257	1.37 (1.04–1.81)	/
Number of risk factors in metabolic health	0	4 (13, 17, 23, 24)	4742405	1.08 (0.92–1.27)	69.77
	≤1	3 (14, 18, 19)	565454	1.03 (0.90–1.18)	53.22
	≤2	1 (26)	25626	1.13 (0.07–1.82)	/
Criteria for defining obesity	IOTF	2 (17, 23)	1156371	0.93 (0.80–1.08)	0.00
	WGOC	2 (18, 19)	560283	0.99 (0.73–1.36)	63.95
	WHO	4 (13, 14, 24, 26)	3616831	1.13 (1.01–1.27)	39.21
Region	Asia	4 (17–19, 23)	1716654	0.98 (0.84–1.14)	61.91
	North America	2 (13, 26)	115883	1.31 (1.03–1.66)	0.00
	Europe	2 (14, 24)	3500948	1.09 (0.94–1.27)	69.77
Proportion of female	<40%	2 (14, 18)	107208	0.95 (0.81–1.12)	6.55
	40%∼90%	4 (17, 19, 24, 27)	5110394	1.08 (1.00–1.18)	63.89
	100%	2 (13, 26)	115883	1.31 (1.03–1.66)	0.00
**MUNW**					
Follow up	<9 years	1 (23)	354083	1.72 (1.55–1.90)	/
	9∼15 years	4 (14, 18, 19, 26)	591080	1.47 (1.23–1.76)	89.68
	>15 years	2 (13, 15)	97379	2.18 (1.87–2.53)	1.36
Number of risk factors in metabolic health	0	2 (13, 23)	444340	1.94 (1.51–2.49)	87.36
	≤1	4 (14, 15, 18, 19)	572576	1.50 (1.25–1.79)	89.58
	≤2	1 (26)	25626	1.24 (0.64–2.40)	/
Criteria for defining obesity	IOTF	1 (23)	354083	1.72 (1.55–1.90)	/
	WGOC	2 (18, 19)	560283	1.64 (1.41–1.91)	80.91
	WHO	4 (13–15, 26)	128176	1.55 (1.00–2.40)	93.62
Region	Asia	3 (18, 19, 27)	914366	1.66 (1.50–1.84)	75.99
	North America	2 (13, 26)	115883	1.82 (1.06–3.13)	65.06
	Europe	2 (14, 15)	12293	1.21 (1.10–1.33)	0.00
Proportion of female	<40%	3 (14, 15, 18)	114330	1.49 (1.06–2.11)	91.77
	40%∼90%	2 (19, 23)	812329	1.61 (1.45–1.79)	74.69
	100%	2 (13, 26)	115883	1.82 (1.06–3.13)	65.06
**MUOW**					
Follow up	<9 years	0	0	/	/
	9∼15 years	3 (18, 19, 26)	585909	1.83 (1.47–2.27)	87.32
	>15 years	1 (13)	90257	2.27 (1.96–2.62)	/
Number of risk factors in metabolic health	0	1 (13)	90257	2.27 (1.96–2.62)	/
	≤1	2 (18, 19)	560283	1.85 (1.44–2.36)	93.64
	≤2	1 (26)	25626	1.74 (1.05–2.88)	/
Criteria for defining obesity	IOTF	0	0	/	/
	WGOC	2 (18, 19)	560283	1.85 (1.44–2.36)	93.64
	WHO	2 (13, 26)	115883	2.22 (1.94–2.56)	0.00
Region	Asia	2 (18, 19)	560283	1.85 (1.44–2.36)	93.64
	North America	2 (13, 26)	115883	2.22 (1.94–2.56)	0.00
	Europe	0	0	/	/
Proportion of female	<40%	1 (18)	102037	2.11 (1.87–2.39)	/
	40%∼90%	1 (19)	458246	1.64 (1.61–1.68)	/
	100%	2 (13, 26)	115883	2.22 (1.94–2.56)	0.00
**MUO**					
Follow up	<9 years	1 (23)	354083	2.06 (1.86–2.29)	/
	9∼15 years	4 (14, 18, 19, 26)	591080	1.83 (1.42–2.36)	92.97
	>15 years	1 (13)	90257	2.58 (2.22–3.00)	/
Number of risk factors in metabolic health	0	2 (13, 23)	444340	2.29 (1.84–2.85)	82.74
	≤1	3 (14, 18, 19)	565454	1.88 (1.43–2.49)	95.28
	≤2	1 (26)	25626	1.49 (0.86–2.58)	/
Criteria for defining obesity	IOTF	1 (23)	354083	2.06 (1.86–2.29)	/
	WGOC	2 (18, 19)	560283	2.11 (1.38–3.22)	97.40
	WHO	3 (13, 14, 26)	121054	1.83 (1.18–2.86)	92.31
Region	Asia	3 (18, 19, 23)	914366	2.09 (1.64–2.66)	95.79
	North America	2 (13, 26)	115883	2.09 (1.24–3.53)	71.98
	Europe	1 (14)	5171	1.49 (1.28–1.74)	/
Proportion of female	<40%	2 (14, 18)	107208	1.98 (1.14–3.46)	96.68
	40%∼90%	2 (19, 23)	812329	1.86 (1.55–2.24)	91.16
	100%	2 (13, 26)	115883	2.09 (1.24–3.53)	71.98

*IOTF, International Obesity Task Force; WGOC, Working Group on Obesity in China; WHO, World Health Organization; MHNW, metabolically healthy normal weight; MHOW, metabolically healthy overweight; MHO, metabolically healthy obese; MUNW, metabolically unhealthy normal weight; MUOW, metabolically unhealthy overweight; MUO, metabolically unhealthy obese.*

#### Metabolically Unhealthy Normal Weight and the Risk of Stroke

**FIGURE 3:**
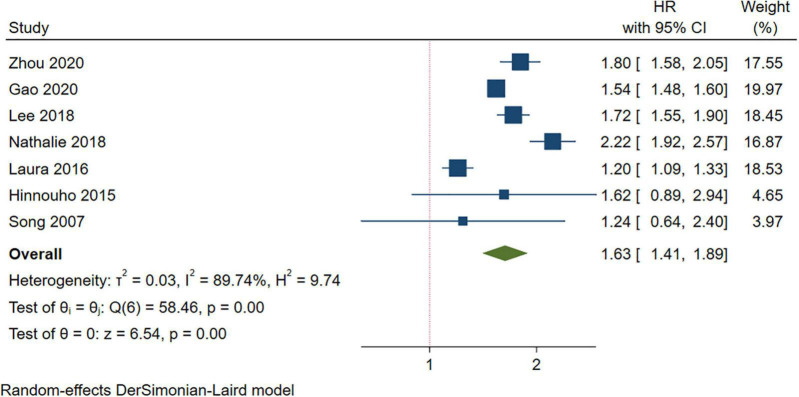
Meta-analysis of the risk of stroke in the MUNW phenotypes compared with the MHNW phenotypes. MUNW, metabolically unhealthy normal weight; MHNW, metabolically healthy normal weight.

A higher risk of stroke was observed in the MUNW group than in the MHNW group (HR = 1.63, 95% CI: 1.41–1.89, *I^2^* = 89.74%, *n* = 7 cohort studies, 1,042,542 participants) (13–15, 18, 19, 23, 26) (Figure 3 and Supplementary Table 2). The comprehensive subgroup analyses (Table 3 and Supplementary Figures 11–15) suggested that the risk of stroke increased when the follow-up was over 15 years (HR = 2.18, 95% CI: 1.87–2.53, *I^2^* = 1.36%) than in those with a shorter follow-up duration, and this meta-analysis was quite homogeneous.

#### Metabolically Unhealthy Overweight/Metabolically Unhealthy Obese and the Risk of Stroke

**FIGURE 4:**
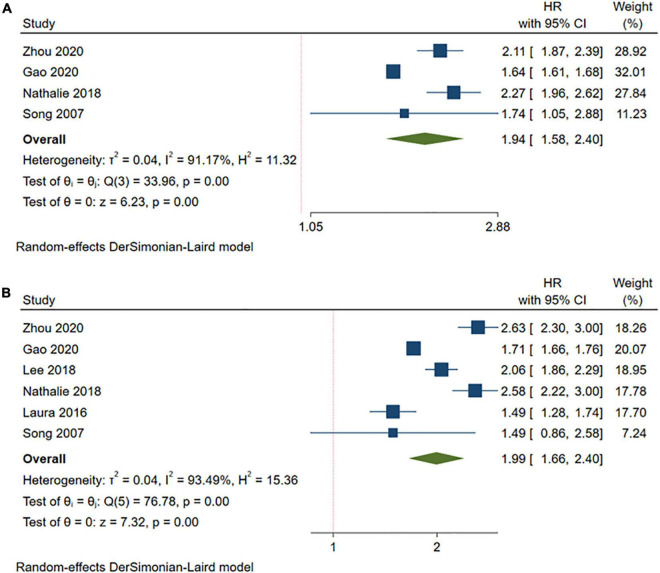
Meta-analysis of the risk of stroke in the MUOW/MUO phenotypes compared with the MHNW phenotypes. **(A)** MUOW phenotypes; **(B)** MUO phenotypes. MHNW, metabolically healthy normal weight; MHOW, metabolically healthy overweight; MHO, metabolically healthy obese; MUNW, metabolically unhealthy normal weight; MUOW, metabolically unhealthy overweight; MUO, metabolically unhealthy obese.

Compared with the MHNW group, a higher risk of stroke was found in either the participants with MUOW (HR = 1.94, 95% CI: 1.58–2.40, *I^2^* = 91.17%, *n* = 4 cohort studies, 676,166 participants) (13, 18, 19, 26) or those with MUO (HR = 1.99, 95% CI: 1.66–2.40, *I^2^* = 93.49%, *n* = 6 cohort studies, 1,035,420 participants) (13, 14, 18, 19, 23, 26) (Figure 4 and Supplementary Table 2). However, the subgroup analyses for the MUOW/MUO studies were substantially heterogeneous, except for the meta-analysis of MUOW only containing studies with a follow-up of more than 15 years, enrolling participants from North America, or studies with females accounting for most of the participants (Table 3 and Supplementary Figures 16–25).

### Sensitivity Analysis and Publication Bias

We performed a sensitivity analysis by omitting only one study with the lowest methodological quality of the NOS (17). An advanced risk of stroke was detected in the MHO participants compared to the MHNW participants (HR = 1.10, 95% CI: 1.03–1.18, *I^2^* = 42.18%, *n* = 7 cohort studies, 4,531,197 participants) (Supplementary Figure 26), which differed from the primary analysis. An additional sensitivity analysis excluding one study at a time is shown in Supplementary Figure 27. The publication bias was not assessed because fewer than ten included studies in each phenotype.

## Discussion

### The Summary of the Main Findings and Strengths Compared With the Previous Studies

This systematic review and meta-analysis including nine prospective cohorts showed that metabolically unhealthy concurrent with either overweight or obesity resulted in an increased risk of stroke, which was not affected by the duration of the follow-up, the number of abnormal metabolic parameters, the definition of obesity, geographic locations, or the proportion of females. Although the primary meta-analysis supported the fact that overweight/obesity with metabolic health did not increase the risk of stroke, some subgroup analyses that only included a specific type of participant might present a different result. This study further examined the causal relationship between overweight/obesity and stroke-onset from the perspective of metabolism, which would assist with the stratified management of overweight/obesity and precision prevention for stroke. Compared with a previous study (16), our study had several strengths in terms of study design and data analyses. First, we included more recent cohorts by an updated search, which improved the precision of the estimated effect size; second, we calculated the HR instead of RR, which considered the time-dependent effect; third, we performed comprehensive subgroup analyses to explore the sources of heterogeneity and to test the robustness of the analysis models.

### Explanations of the Results and Clinical Implications

We found that individuals with metabolically unhealthy phenotypes were at a higher risk of stroke, irrespective of the body weight, which was consistent with a previous systematic review (14, 16, 23). This suggests that multiple coexisting metabolic parameters play a leading role in the risk of incident stroke.

However, regarding the phenotypes of MHOW and MHO, our study suggested that the associated risk of stroke was not significantly increased, which differed from a previous systematic review (16). This was partially explained by the updates of the literature search and the different types of outcome data included in the meta-analysis. Furthermore, we observed additional findings when comprehensive subgroup analyses were performed. Concerning the participants with MHOW, a higher risk of stroke was identified by a longer duration of follow-up. This could be explained by the transition of the metabolic health status over long term. As studies have shown, MHOW is probably an unstable and intermediate condition, which could transfer to a metabolically unhealthy phenotype during the follow-up (28). Regarding the subgroup analysis of MHO, we found that MHO led to a higher risk of stroke only when the participants were all females and living in North America. This could be partially explained by the sex differences in the development of stroke. It has been well-recognized that the incidence of stroke is higher in women than in men after the menopausal period (29). The subgroup enrolled middle-aged female participants and continued the follow-up for incident stroke for more than 10 years immediately after their menopausal period. Another reason for the outstanding risk of stroke in the subgroup could be the high transition rate from metabolic health to unhealth in women. The Nurses’ Health Study reported that 84% of the women with MHO and 68% with MHNW were converted to metabolically unhealthy (13). The subgroup analysis also identified an associated risk of stroke for the MHO participants with obesity diagnosed based on the WHO criteria. This was not unexpected because the WHO defined obesity as a BMI of >30 kg/m^2^ (30), which was higher than any other criteria, such as the IOTF and the WGOC standards.

This study has several potential implications in clinical practice. First, metabolically unhealthy and elevated body weight had combined effects on increasing the risk of stroke. This suggests that when a strategy for stroke prevention is developed, monitoring metabolic parameters and achieving optimal body weight should be highlighted. Second, the potential long-term risk of stroke in individuals with metabolic health overweight/obesity should not be neglected, as the metabolic status might change over time, particularly in women. This suggests that maintaining metabolic health is also a goal for individuals with elevated body weight in the primary prevention of stroke. Third, as the homogeneous subgroup analysis showed, women appeared to have a higher risk of stroke when they had MHO, MUOW, and MUO. This suggests that the stroke prevention strategies tailored to female individuals with elevated body weight should be further studied and developed.

### Limitations

This study has limitations. First, a few of the included cohorts performed multiple measurements of metabolic parameters during the follow-up, which might underestimate the impact of metabolic transitions on the risk of stroke. Second, our meta-analysis only calculated the risk of all stroke events, as most studies did not specify the type of stroke, and the confirmation method of the stroke events varied. This may not be sufficient to develop a precise prevention strategy for a specific type of stroke. Third, pooled HRs in this study were estimated by the random model to identify the average effect and the overall trend, therefore, although the statistical results potentially referred to a causal relationship of moderate-to-high strength, the HR value should not be considered as a typical risk effect (31, 32). Fourth, the definition of metabolic health varies, which may be changed alongside the ongoing studies. This suggests that an updated meta-analysis of the best available evidence is needed (33, 34). Finally, we only searched three representative English literature databases, which may have omitted some additional studies. However, methodological studies have suggested that restricting systematic reviews to English-language publications appears to have little impact on the effect estimates and conclusions of systematic reviews (35, 36).

## Conclusion

The risk of stroke increases for all metabolically unhealthy phenotypes, irrespective of the BMI. The associated risk of stroke with metabolic health, but high BMI, is at substantial heterogeneity, which requires future research considering the impact of sex and transition of the metabolic status on the risk of stroke.

## Data Availability Statement

The original contributions presented in the study are included in the article/Supplementary Material, further inquiries can be directed to the corresponding author/s.

## Author Contributions

MM conceived the manuscript and analyzed the data. MM and YG searched the literature and collected the data. LL, ZK, and MM evaluated the methodological quality. XN and YC interpreted the results. XN designed the study, supervised the study implementation, validated the data, and wrote the manuscript. All authors approved the final manuscript.

## Conflict of Interest

The authors declare that the research was conducted in the absence of any commercial or financial relationships that could be construed as a potential conflict of interest.

## Publisher’s Note

All claims expressed in this article are solely those of the authors and do not necessarily represent those of their affiliated organizations, or those of the publisher, the editors and the reviewers. Any product that may be evaluated in this article, or claim that may be made by its manufacturer, is not guaranteed or endorsed by the publisher.

## Supplementary Material

Supplementary Data Sheet 1 The full list of excluded studies.

## Abbreviations

BMI: body mass index
MHNW: metabolically healthy normal weight
MHOW: metabolically healthy overweight
MHO: metabolically healthy obese
MUNW: metabolically unhealthy normal weight
MUOW: metabolically unhealthy overweight
MUO: metabolically unhealthy obese
IOTF: International Obesity Task Force
WGOC: Working Group on Obesity in China
WHO: World Health Organization.
