# Supplementary material for: Risk of Stroke Among Different Metabolic Obesity Phenotypes: A Systematic Review and Meta-Analysis
Source: Front Cardiovasc Med. 2022 Apr 25;9:844550. doi: 10.3389/fcvm.2022.844550 (PMC9081493; doi:10.3389/fcvm.2022.844550)
Supplement: Supplementary file 2 [file Data_Sheet_2.pdf]

Supplementary Figures

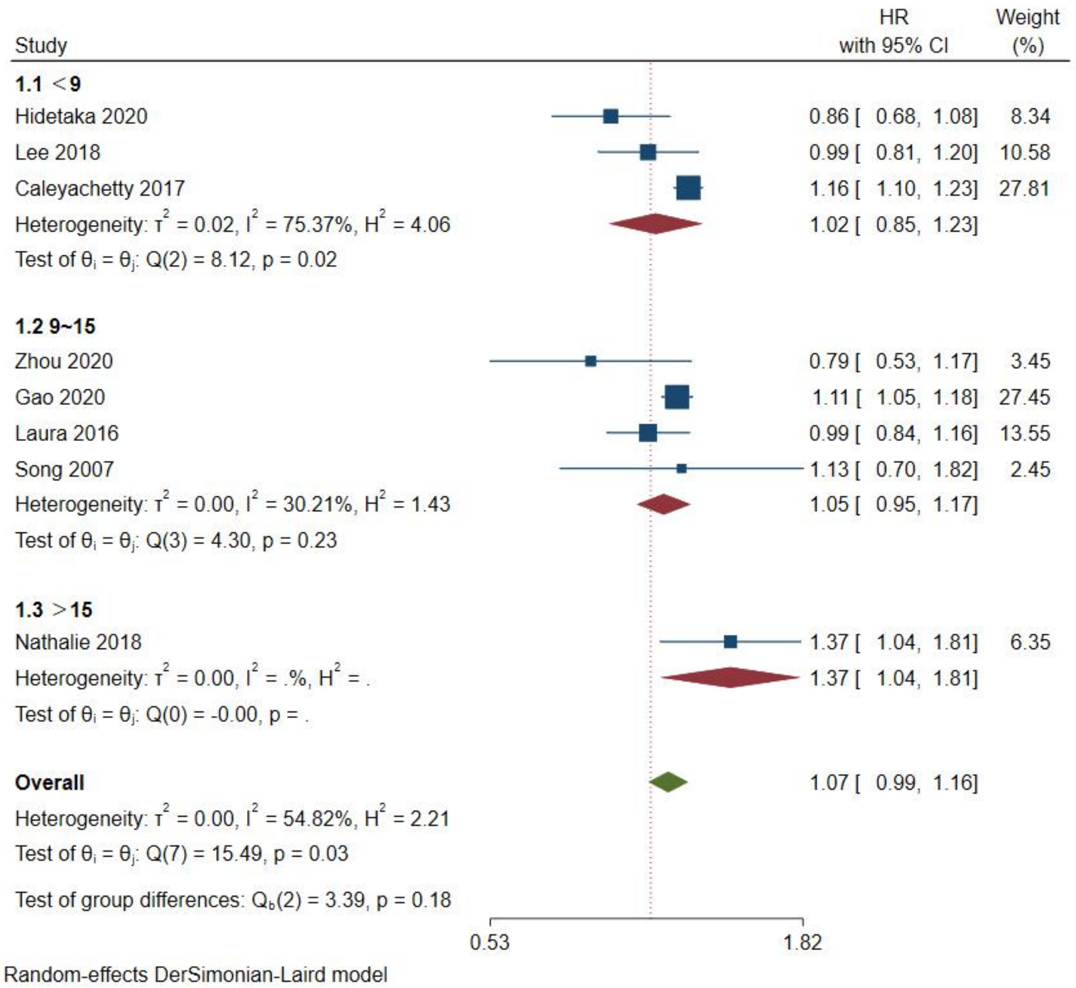

Figure S1 Subgroup (follow up) analysis of risk of stroke in MHO phenotypes compared with MHNW phenotypes

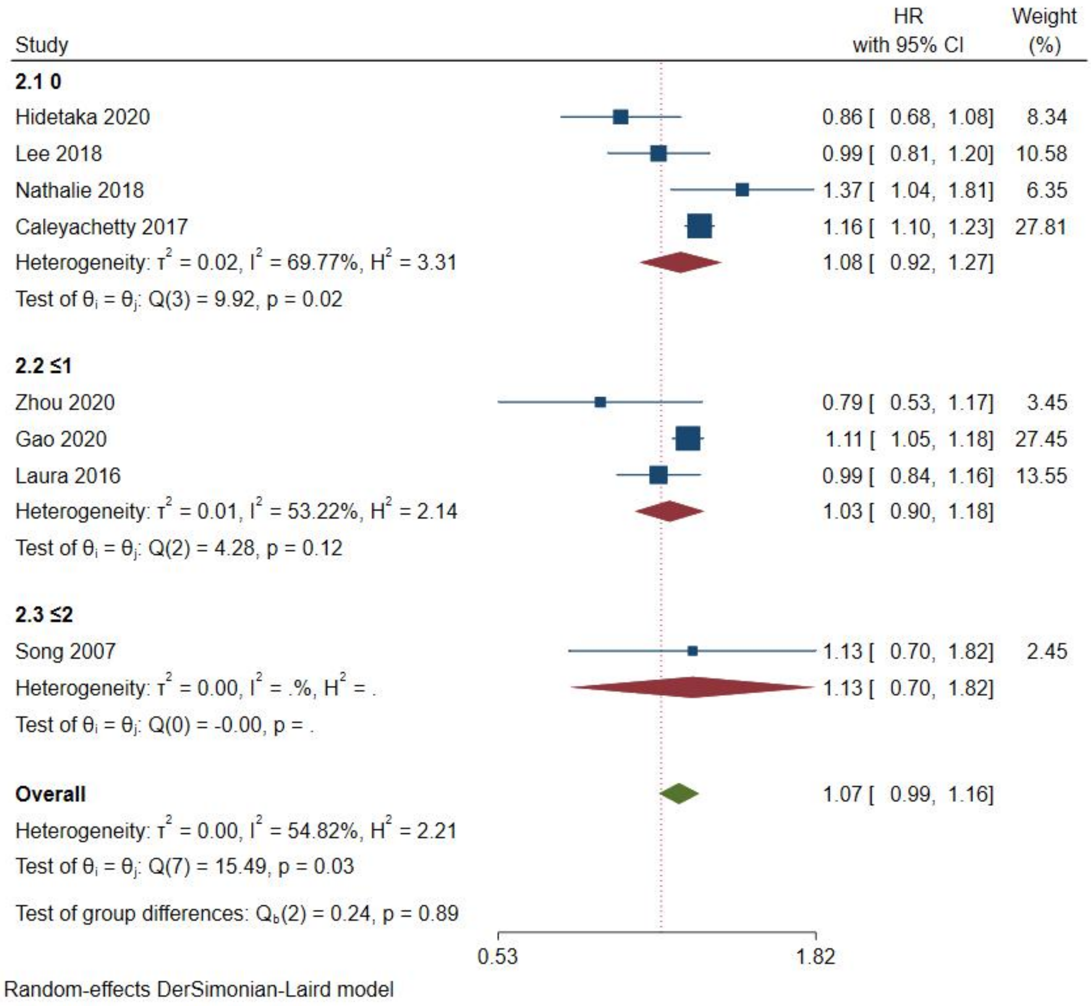

Figure S2 Subgroup (number of risk factors in metabolic health) analysis of risk of stroke in MHO phenotypes compared with MHNW phenotypes

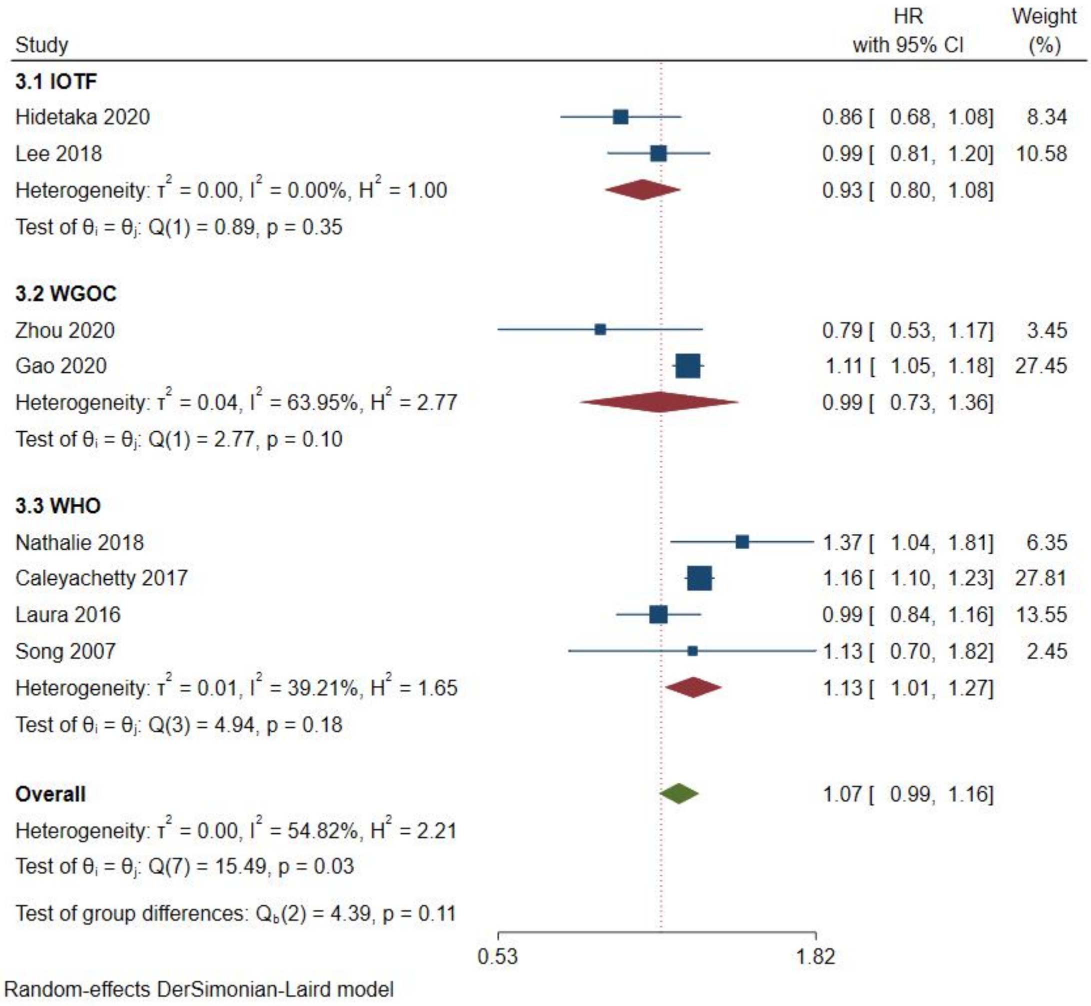

Figure S3 Subgroup (criteria for defining obesity) analysis of risk of stroke in MHO phenotypes compared with MHNW phenotypes

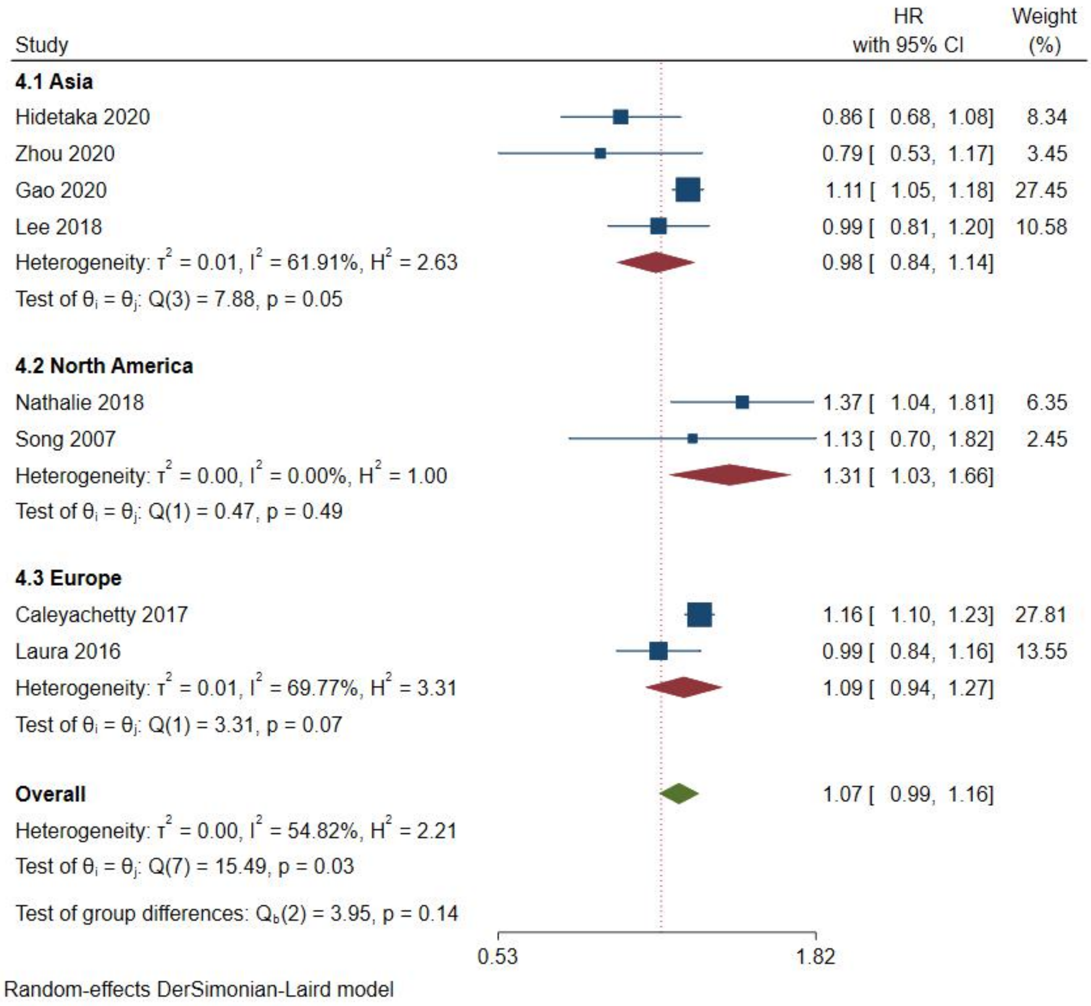

Figure S4 Subgroup (region) analysis of risk of stroke in MHO phenotypes compared with MHNW phenotypes

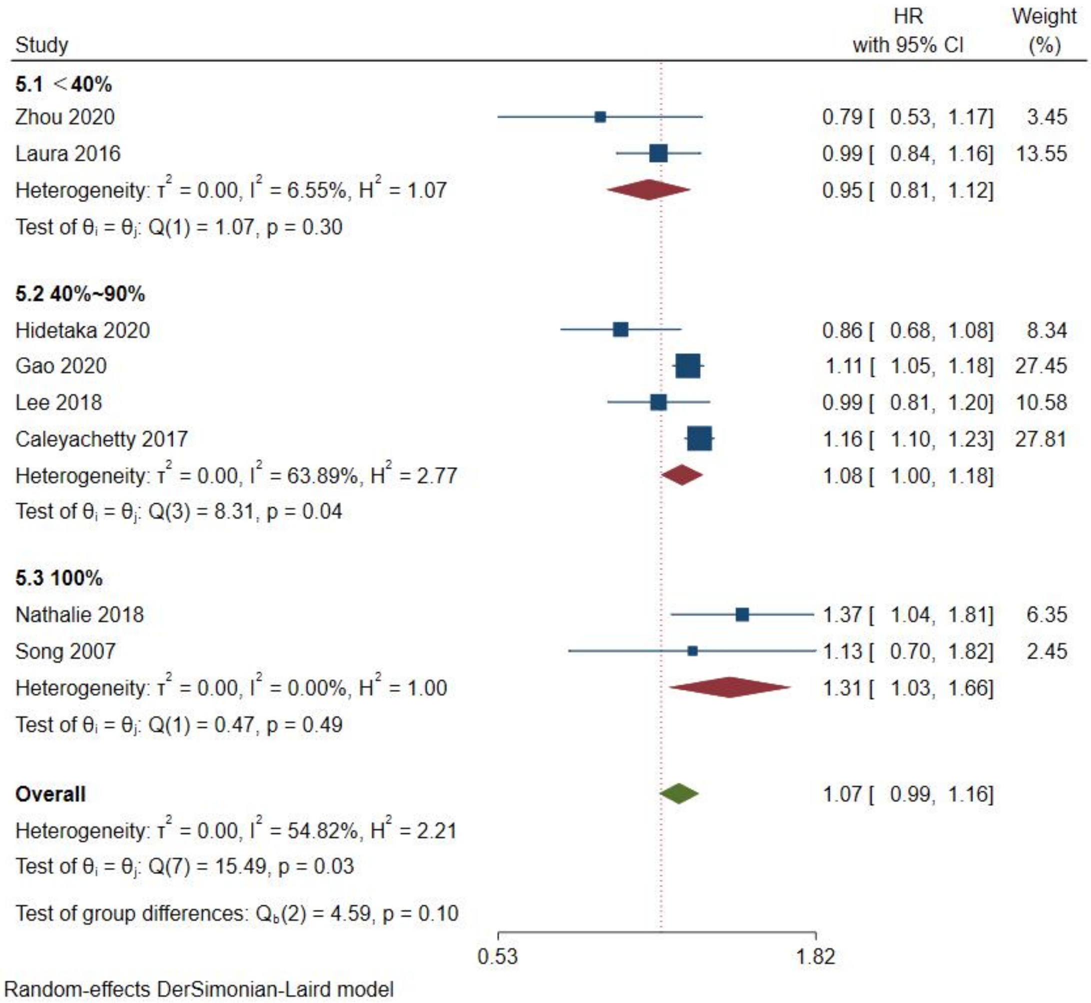

Figure S5 Subgroup (proportion of female) analysis of risk of stroke in MHO phenotypes compared with MHNW phenotypes

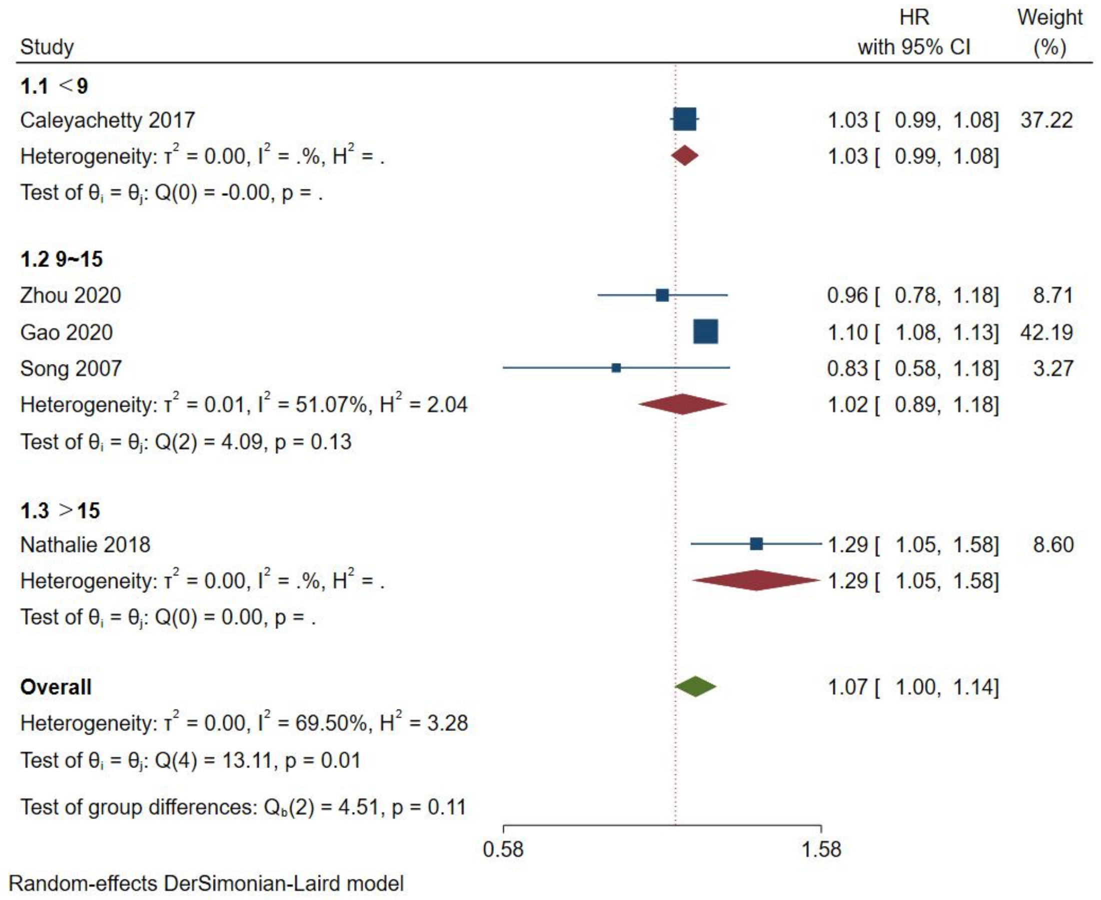

Figure S6 Subgroup (follow up) analysis of risk of stroke in MHOW phenotypes compared with MHNW phenotypes

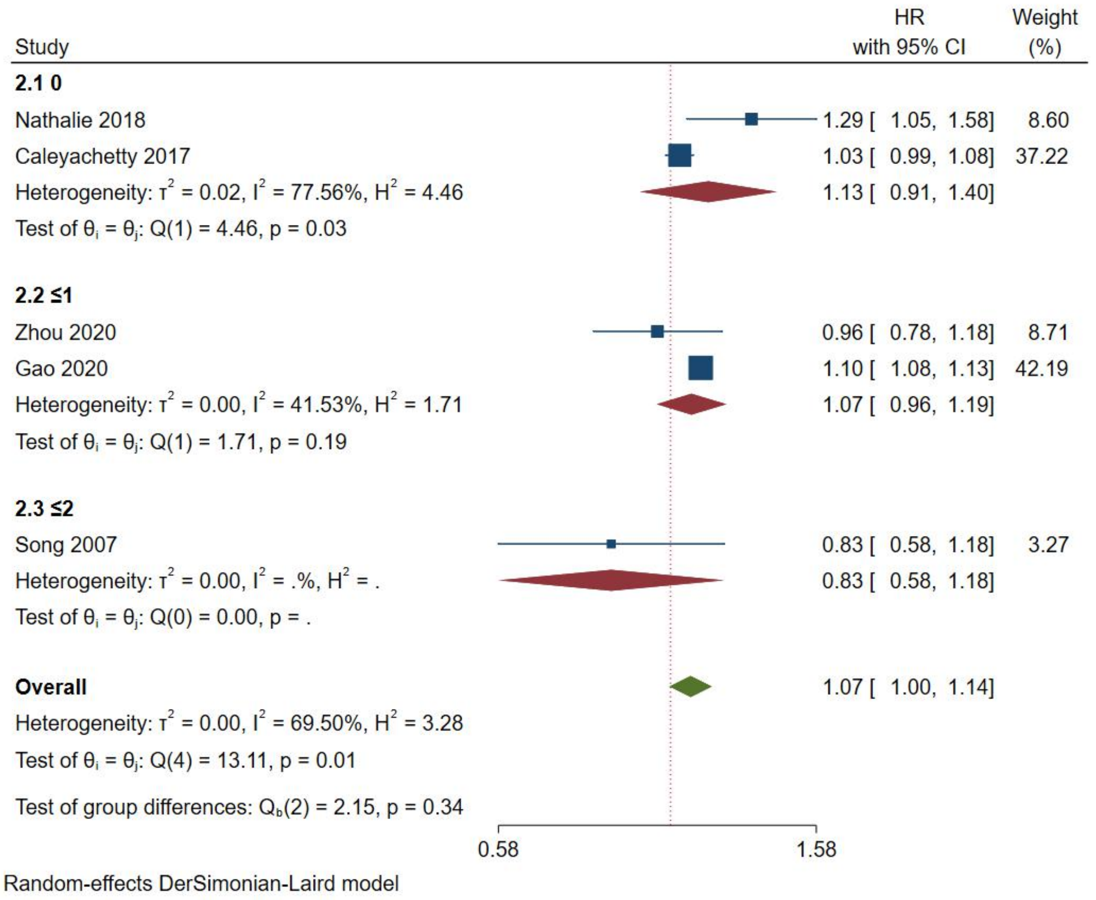

Figure S7 Subgroup (number of risk factors in metabolic health) analysis of risk of stroke in MHOW phenotypes compared with MHNW phenotypes

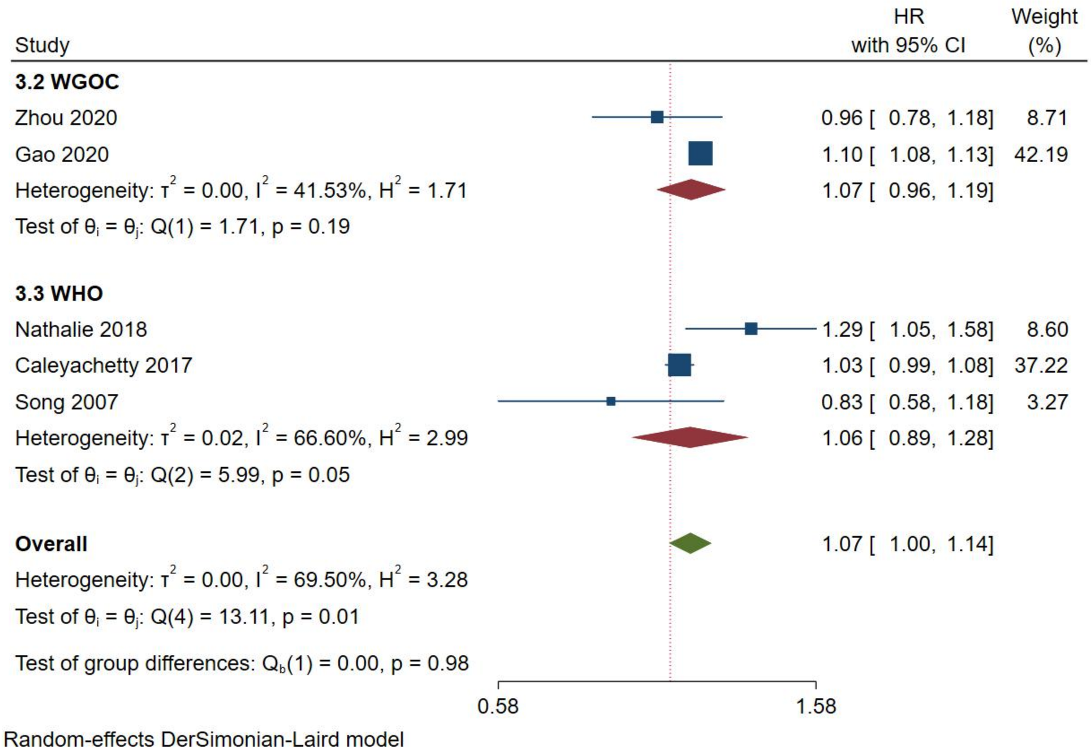

Figure S8 Subgroup (criteria for defining obesity) analysis of risk of stroke in MHOW phenotypes compared with MHNW phenotypes

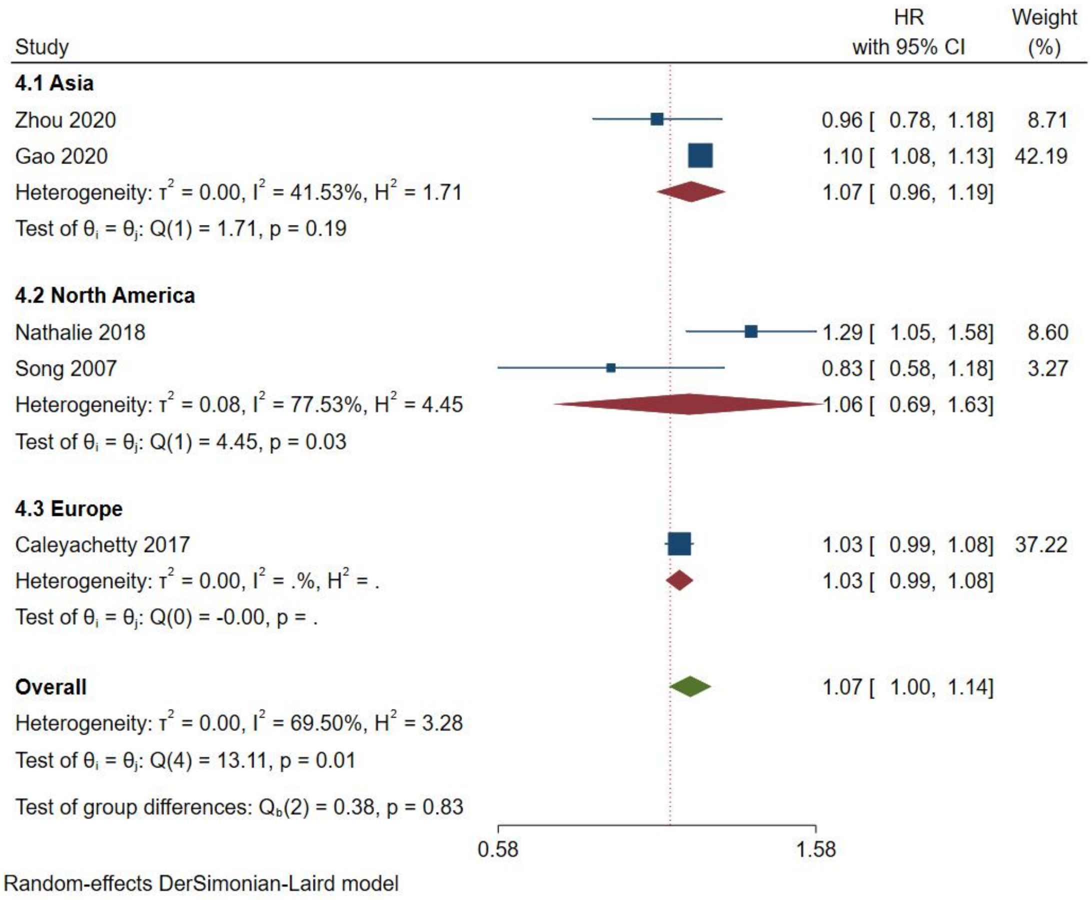

Figure S9 Subgroup (region) analysis of risk of stroke in MHOW phenotypes compared with MHNW phenotypes

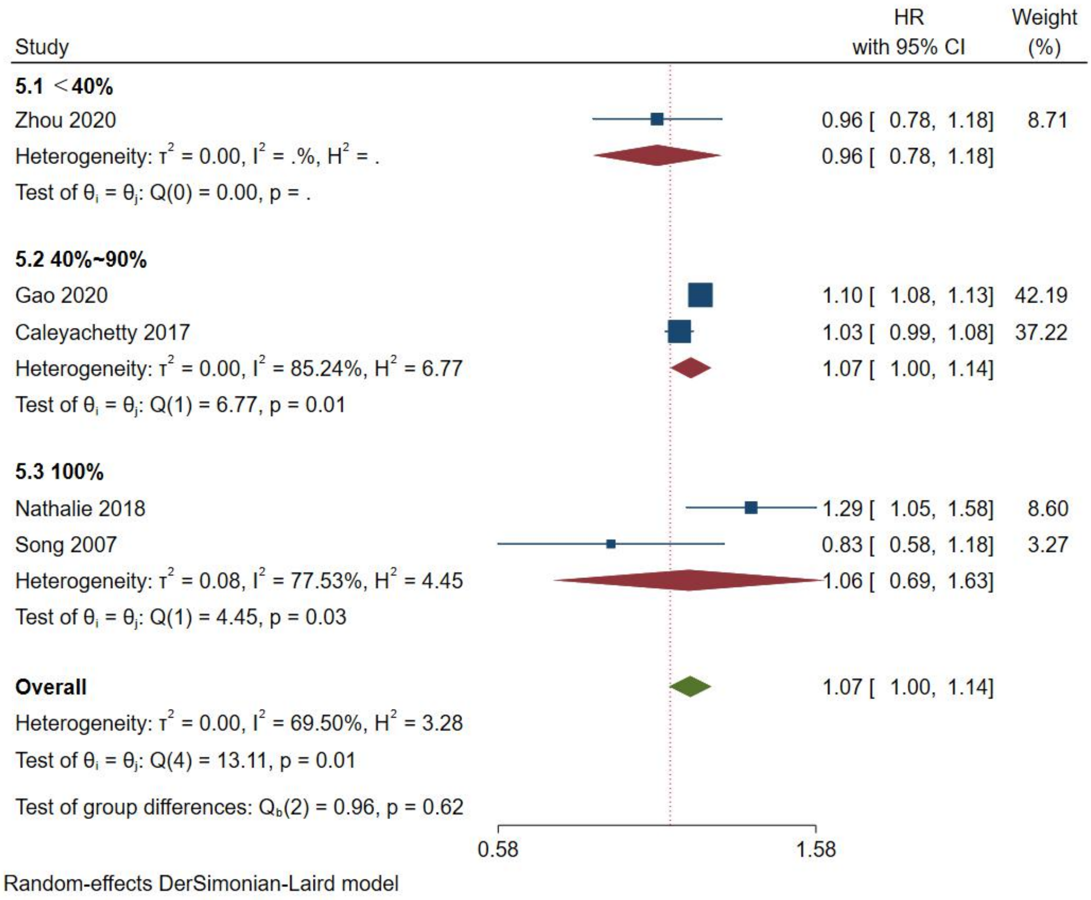

Figure S10 Subgroup (proportion of female) analysis of risk of stroke in MHOW phenotypes compared with MHNW phenotypes

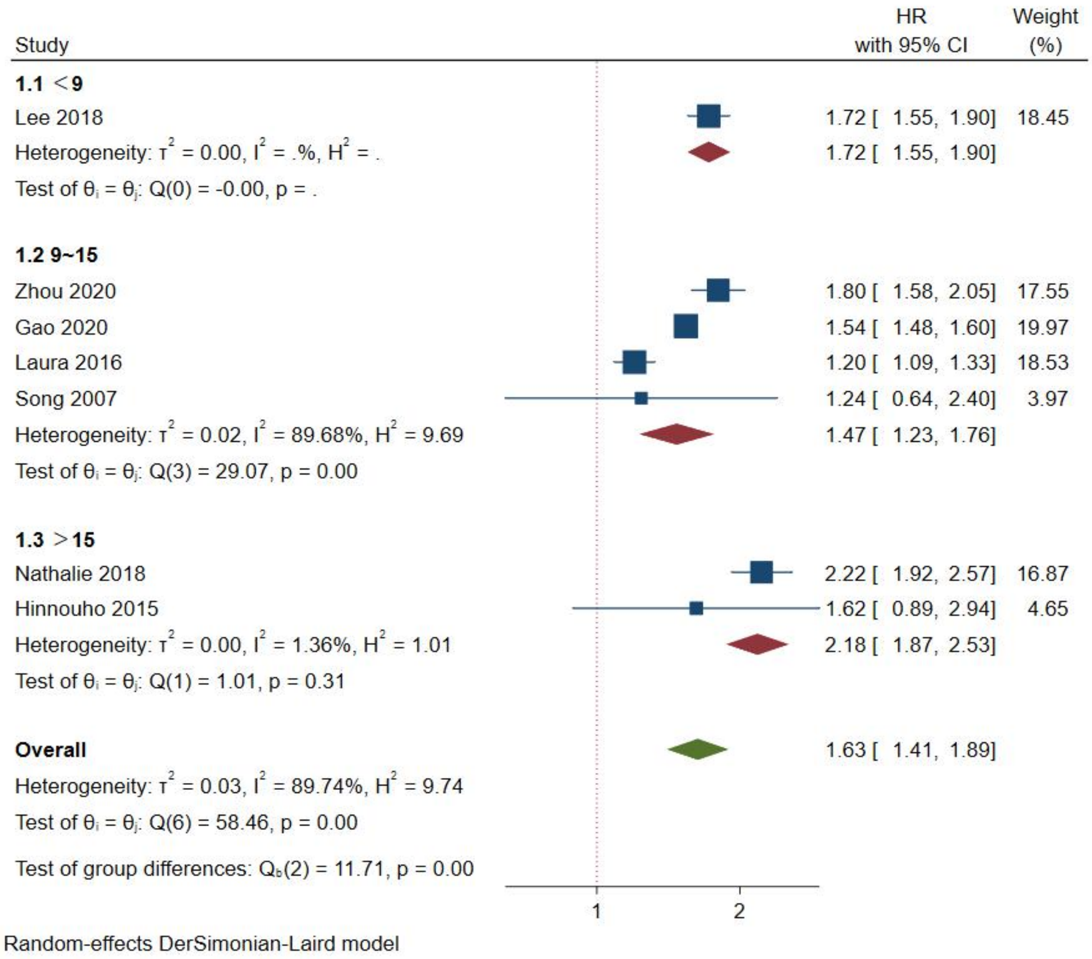

Figure S11 Subgroup (follow up) analysis of risk of stroke in MUNW phenotypes compared with MHNW phenotypes

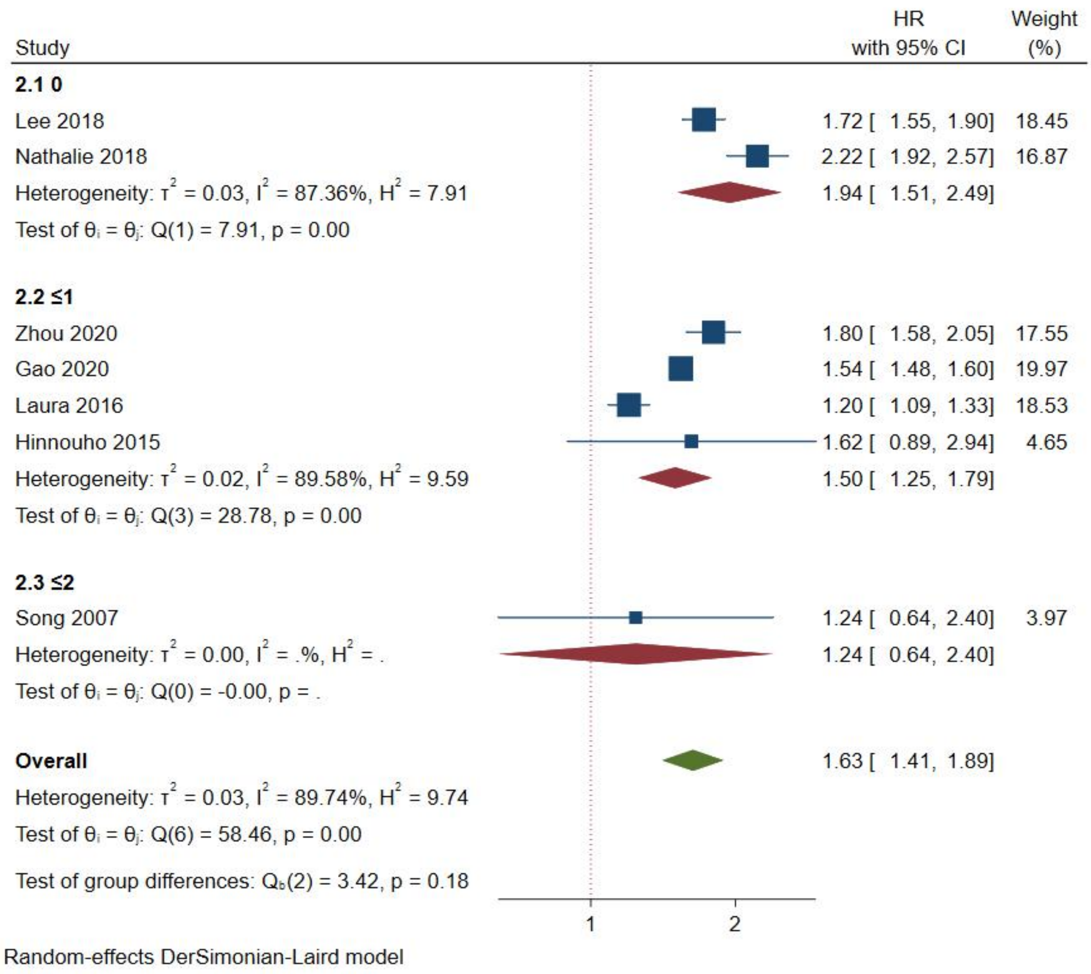

Figure S12 Subgroup (number of risk factors in metabolic health) analysis of risk of stroke in MUNW phenotypes compared with MHNW phenotypes

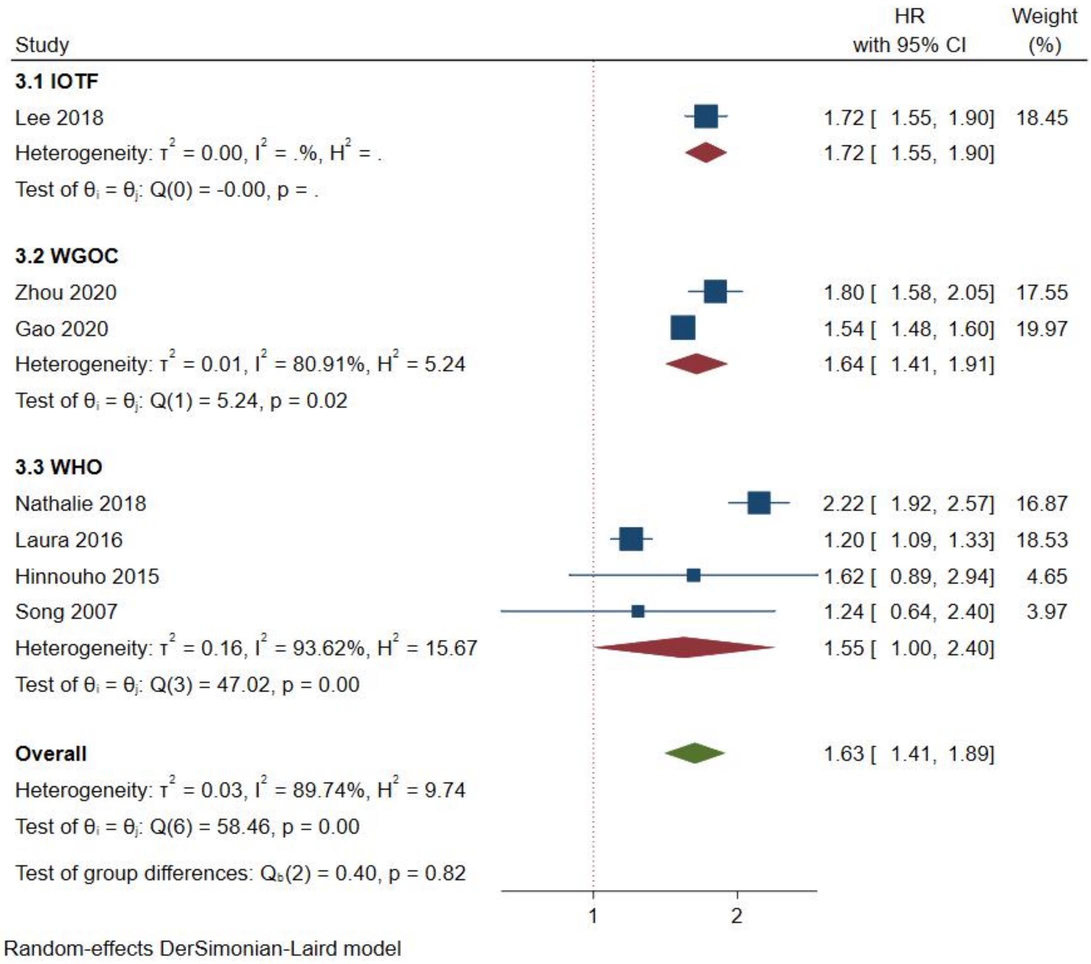

Figure S13 Subgroup (criteria for defining obesity) analysis of risk of stroke in MUNW phenotypes compared with MHNW phenotypes

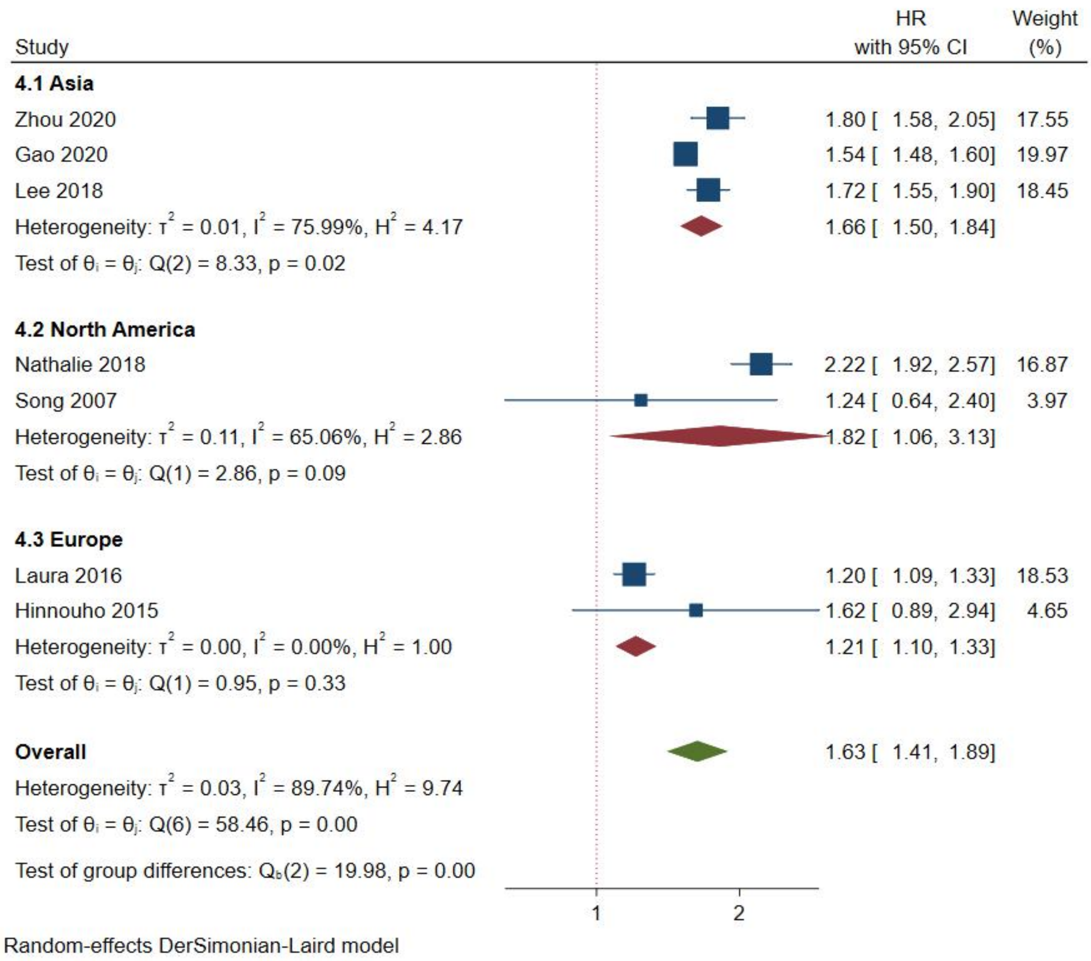

Figure S14 Subgroup (region) analysis of risk of stroke in MUNW phenotypes compared with MHNW phenotypes

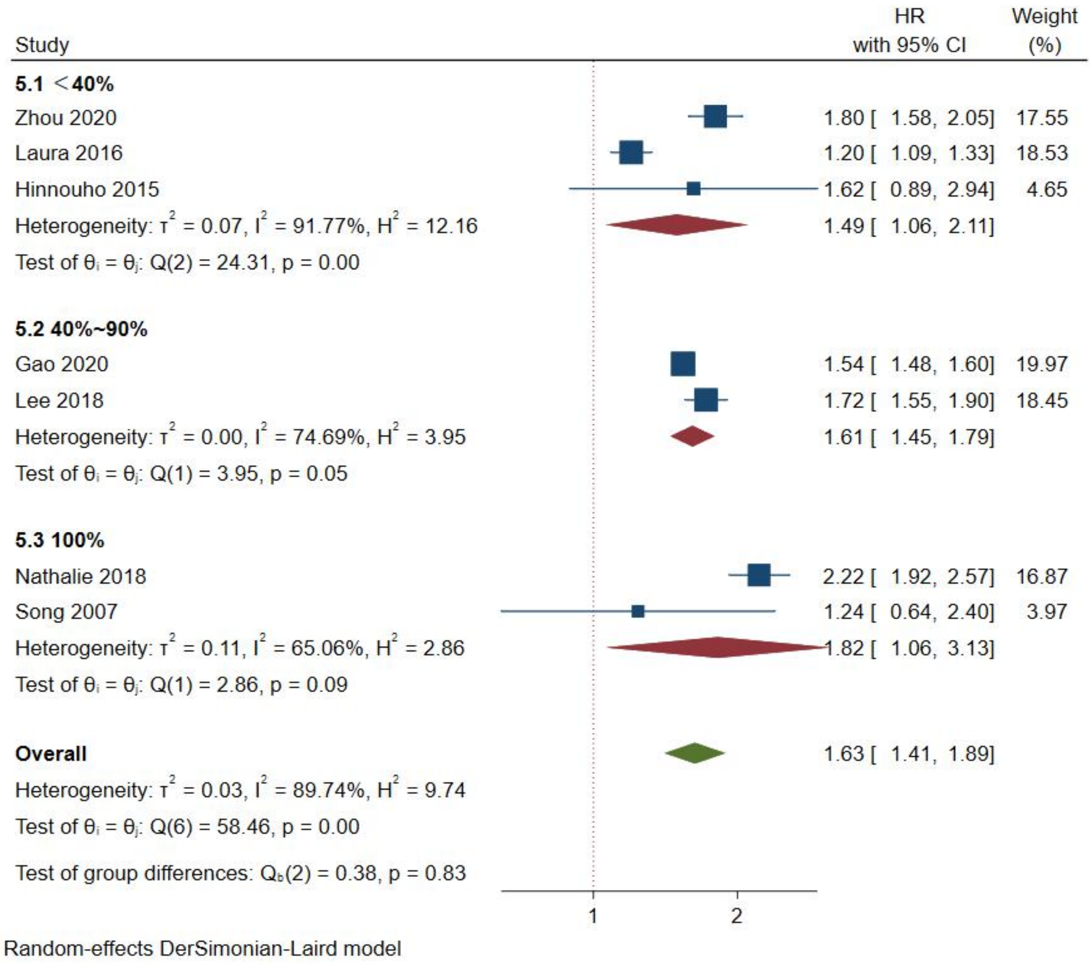

Figure S15 Subgroup (proportion of female) analysis of risk of stroke in MUNW phenotypes compared with MHNW phenotypes

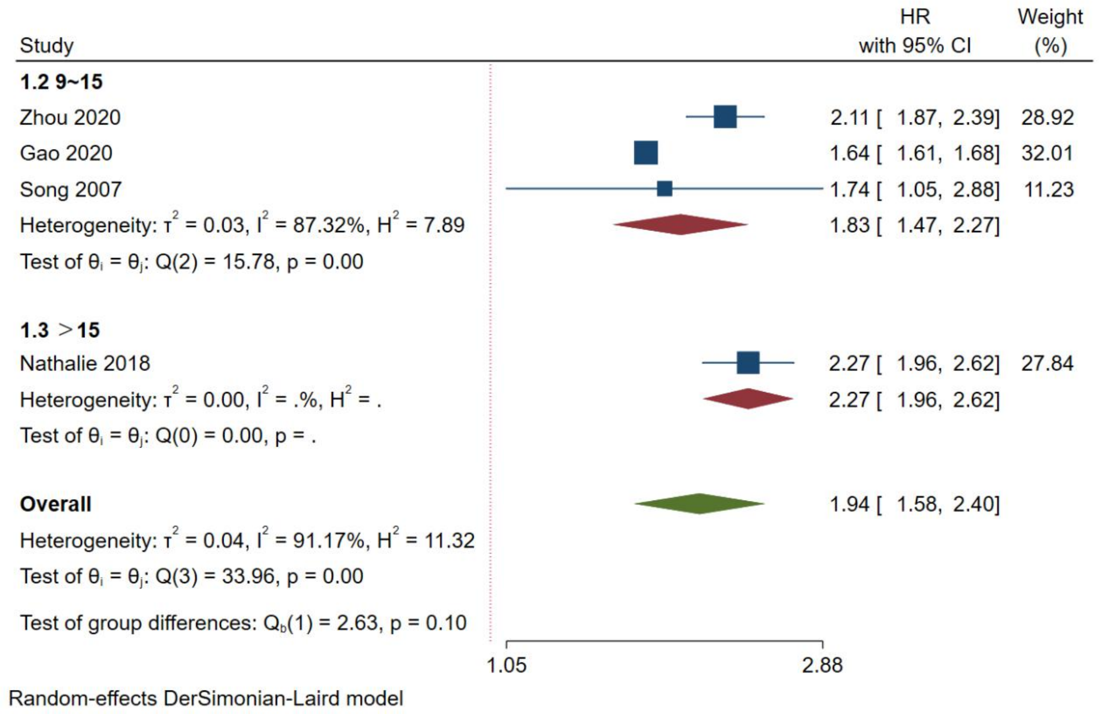

Figure S16 Subgroup (follow up) analysis of risk of stroke in MUOW phenotypes compared with MHNW phenotypes

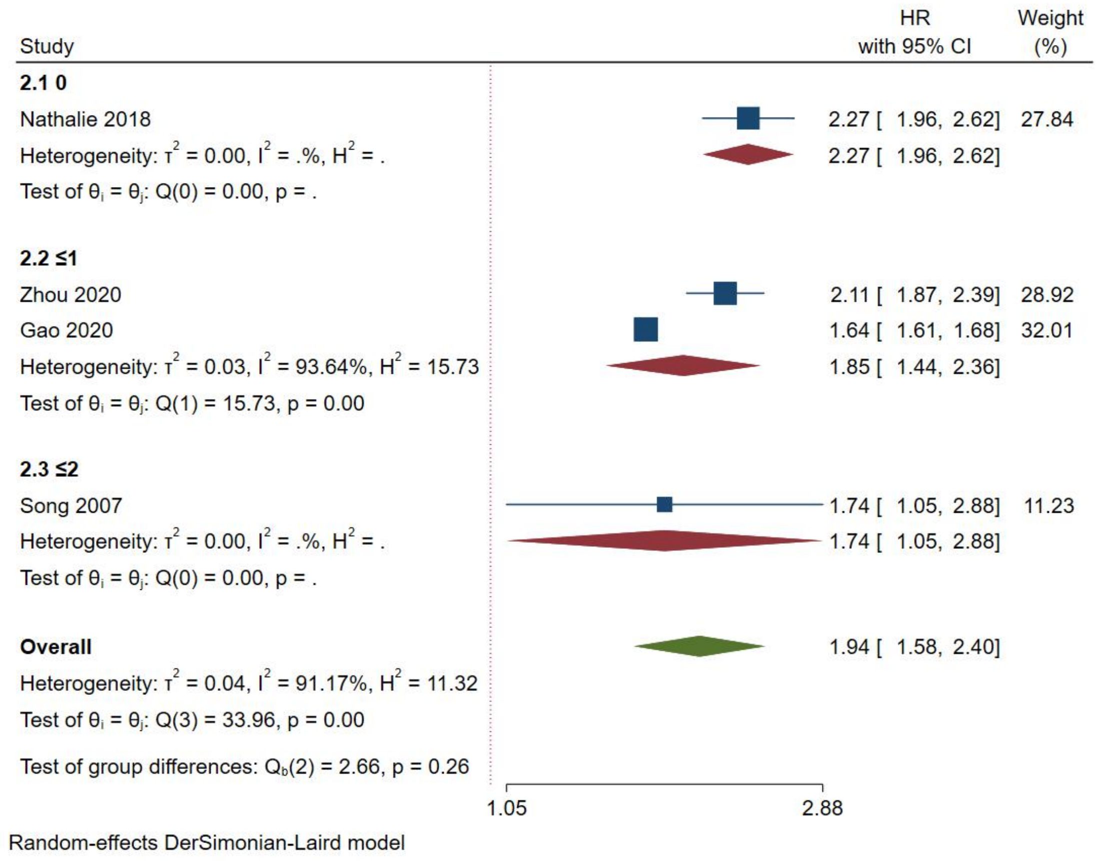

Figure S17 Subgroup (number of risk factors in metabolic health) analysis of risk of stroke in MUOW phenotypes compared with MHNW phenotypes

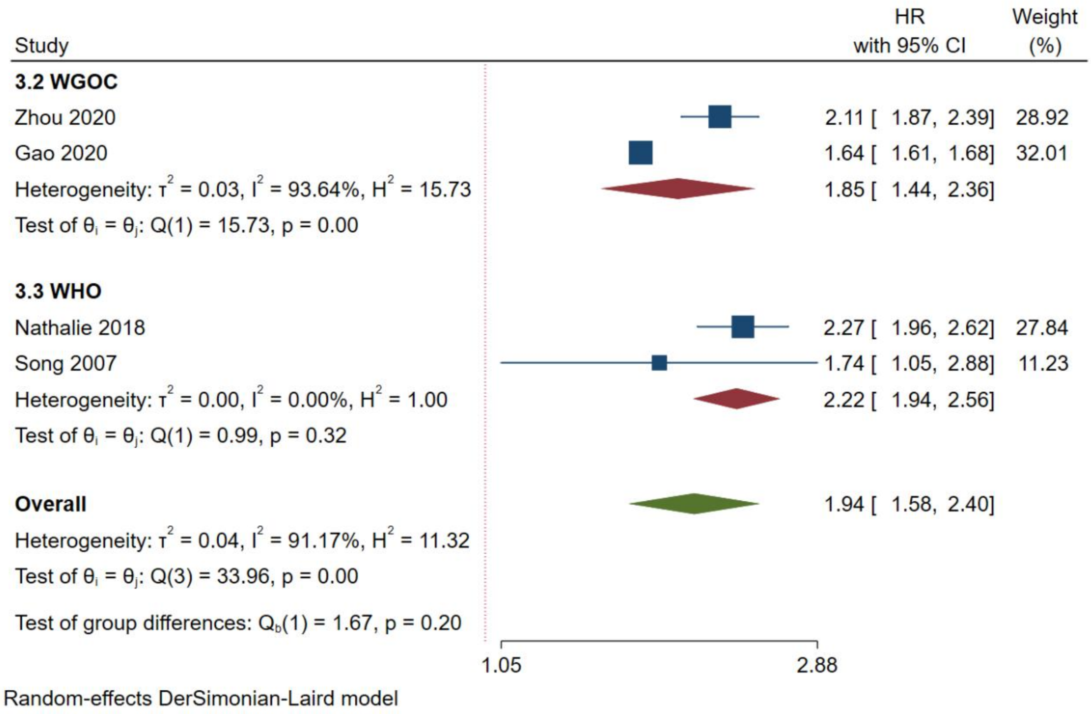

Figure S18 Subgroup (criteria for defining obesity) analysis of risk of stroke in MUOW phenotypes compared with MHNW phenotypes

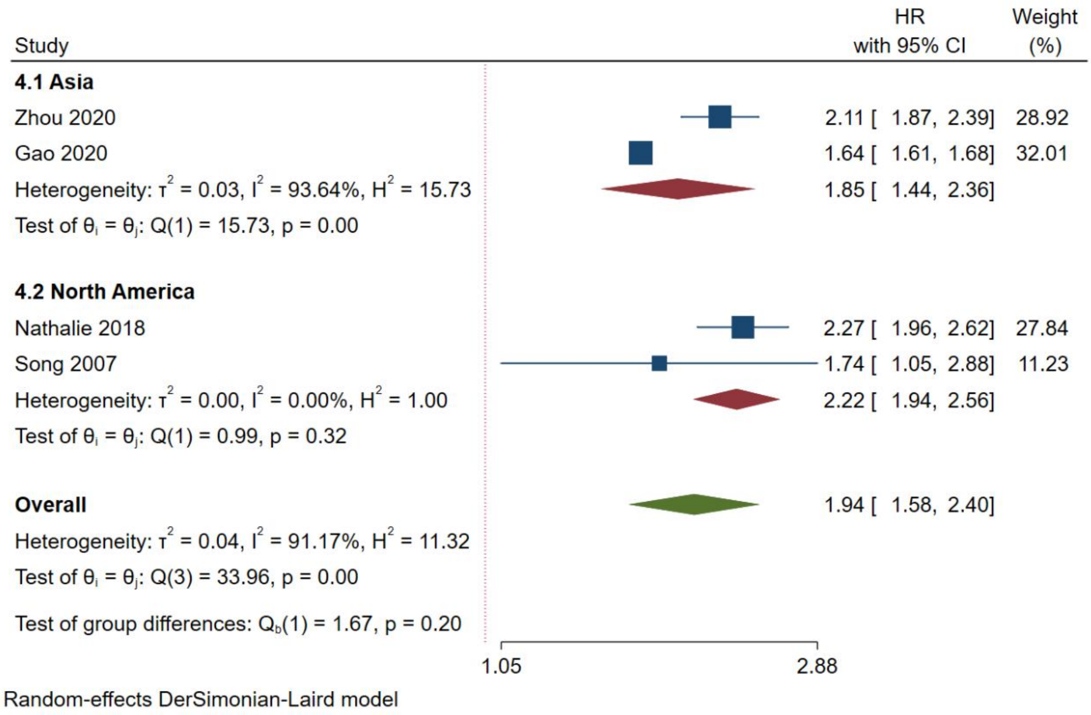

Figure S19 Subgroup (region) analysis of risk of stroke in MUOW phenotypes compared with MHNW phenotypes

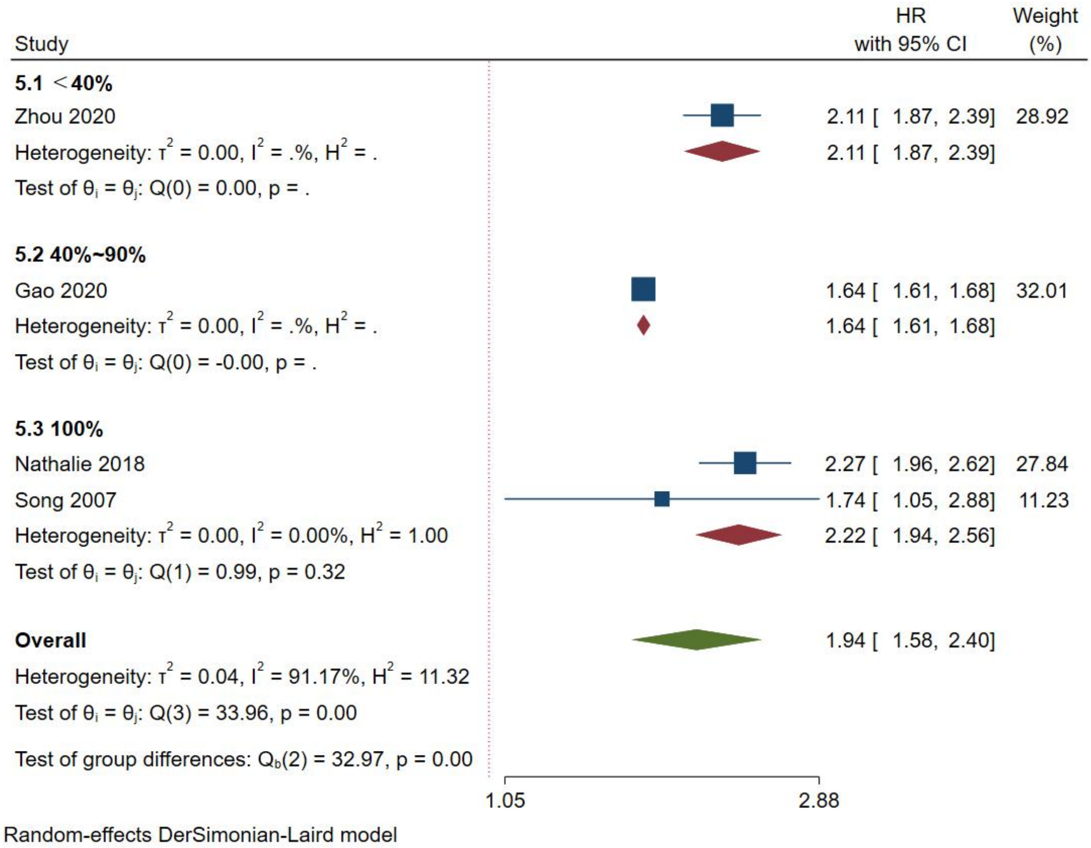

Figure S20 Subgroup (proportion of female) analysis of risk of stroke in MUOW phenotypes compared with MHNW phenotypes

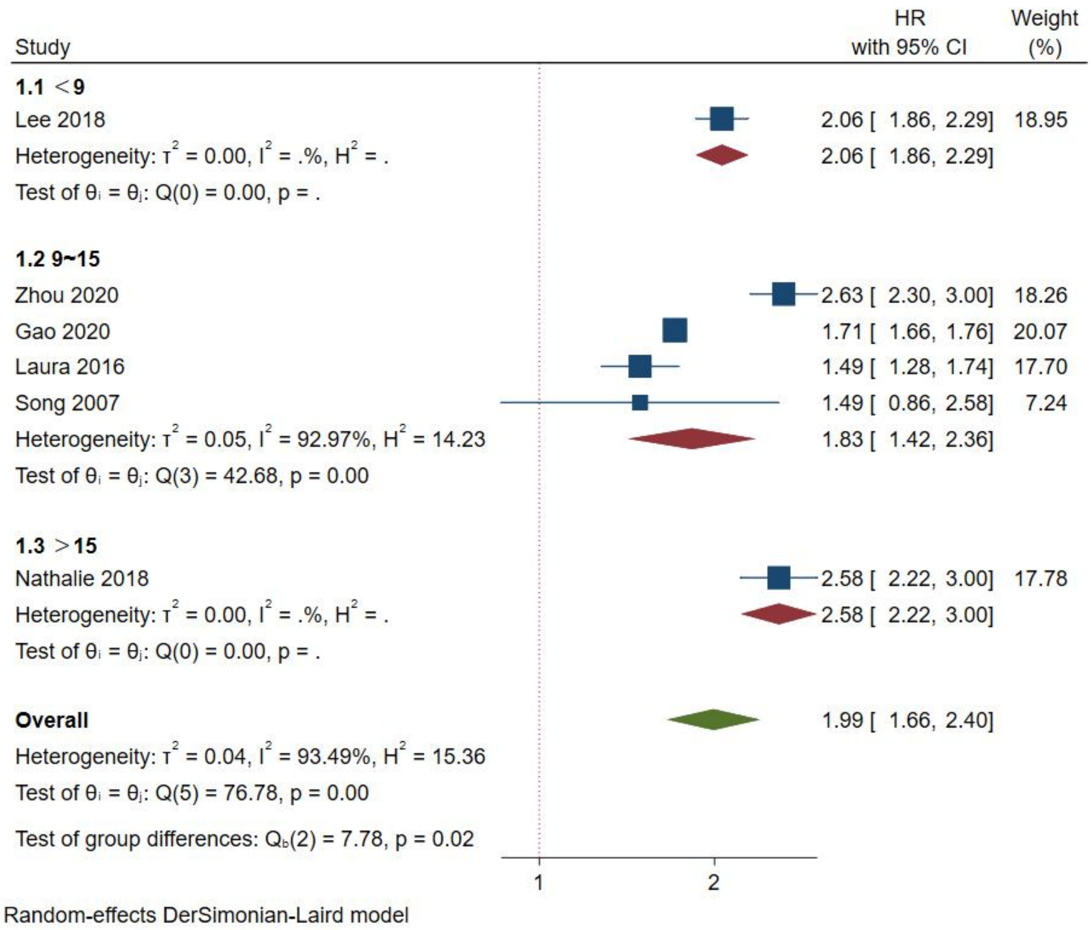

Figure S21 Subgroup (follow up) analysis of risk of stroke in MUO phenotypes compared with MHNW phenotypes

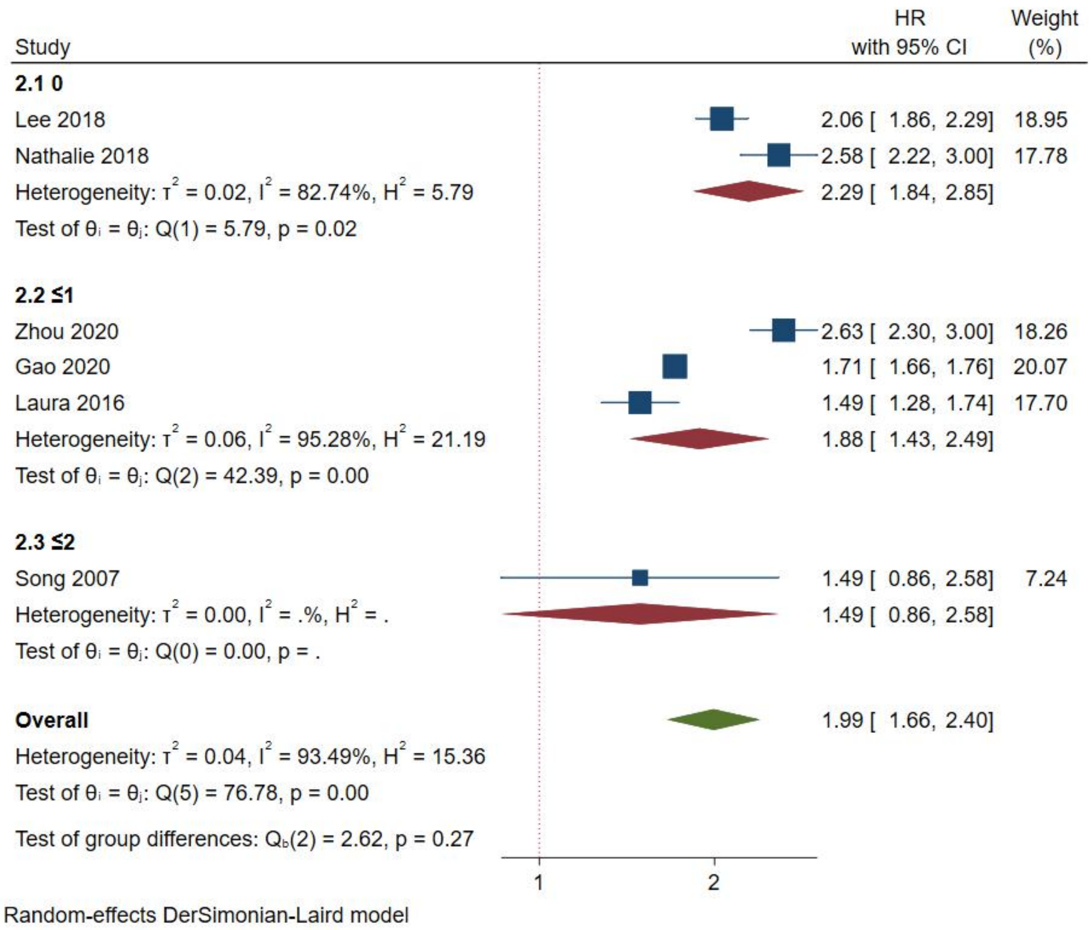

Figure S22 Subgroup (number of risk factors in metabolic health) analysis of risk of stroke in MUO phenotypes compared with MHNW phenotypes

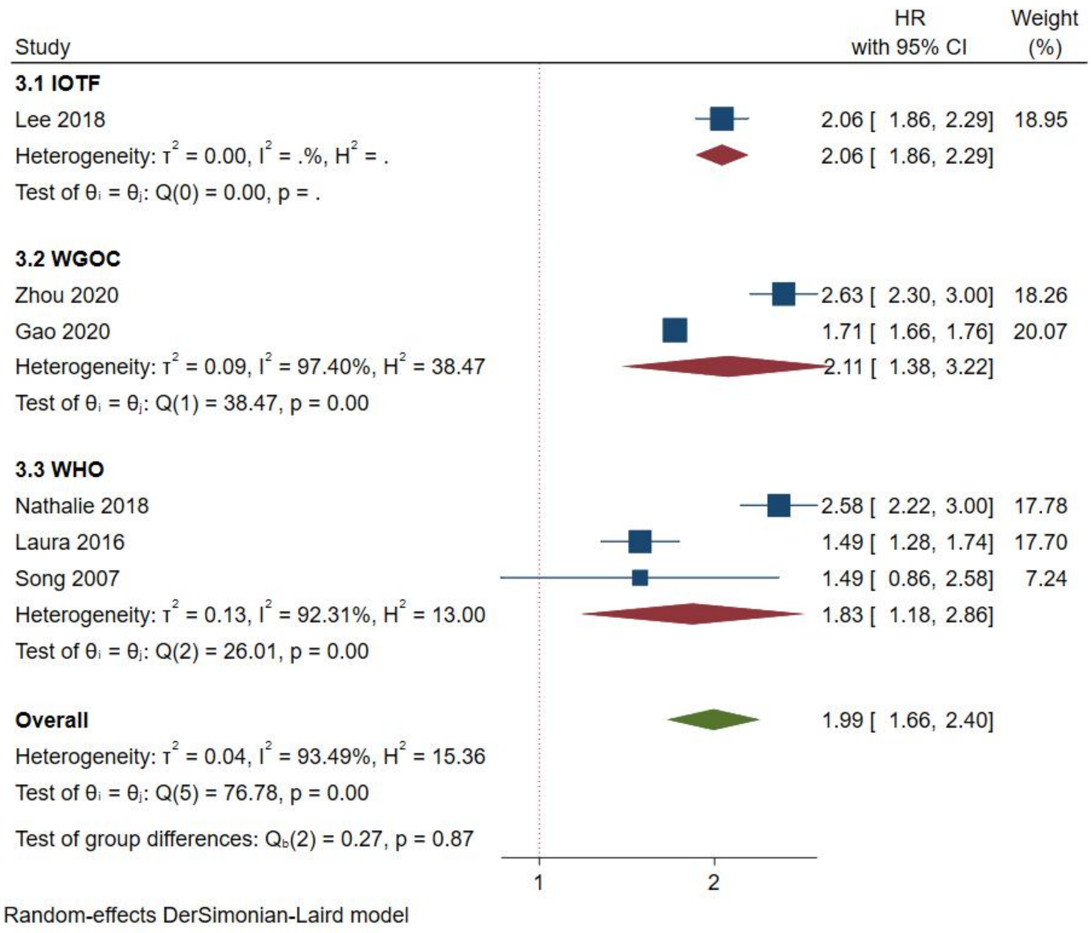

Figure S23 Subgroup (criteria for defining obesity) analysis of risk of stroke in MUO phenotypes compared with MHNW phenotypes

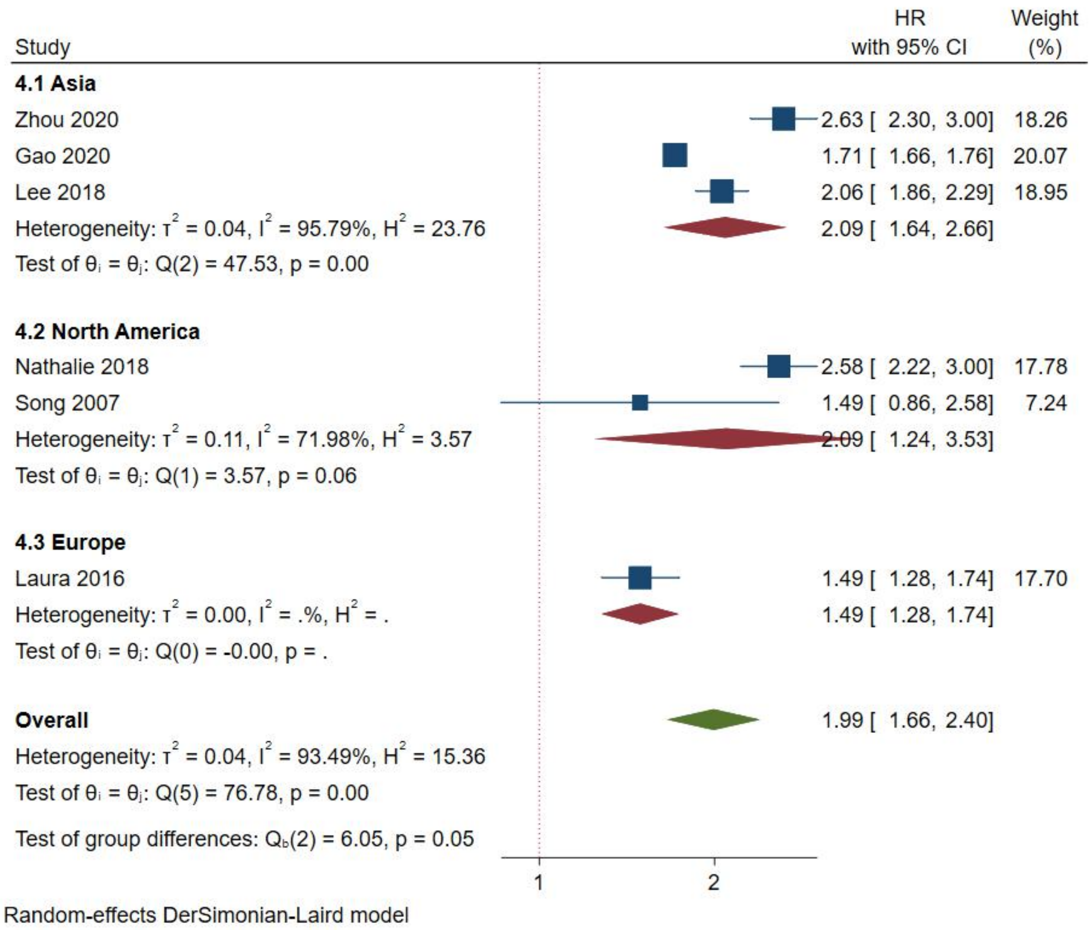

Figure S24 Subgroup (region) analysis of risk of stroke in MUO phenotypes compared with MHNW phenotypes

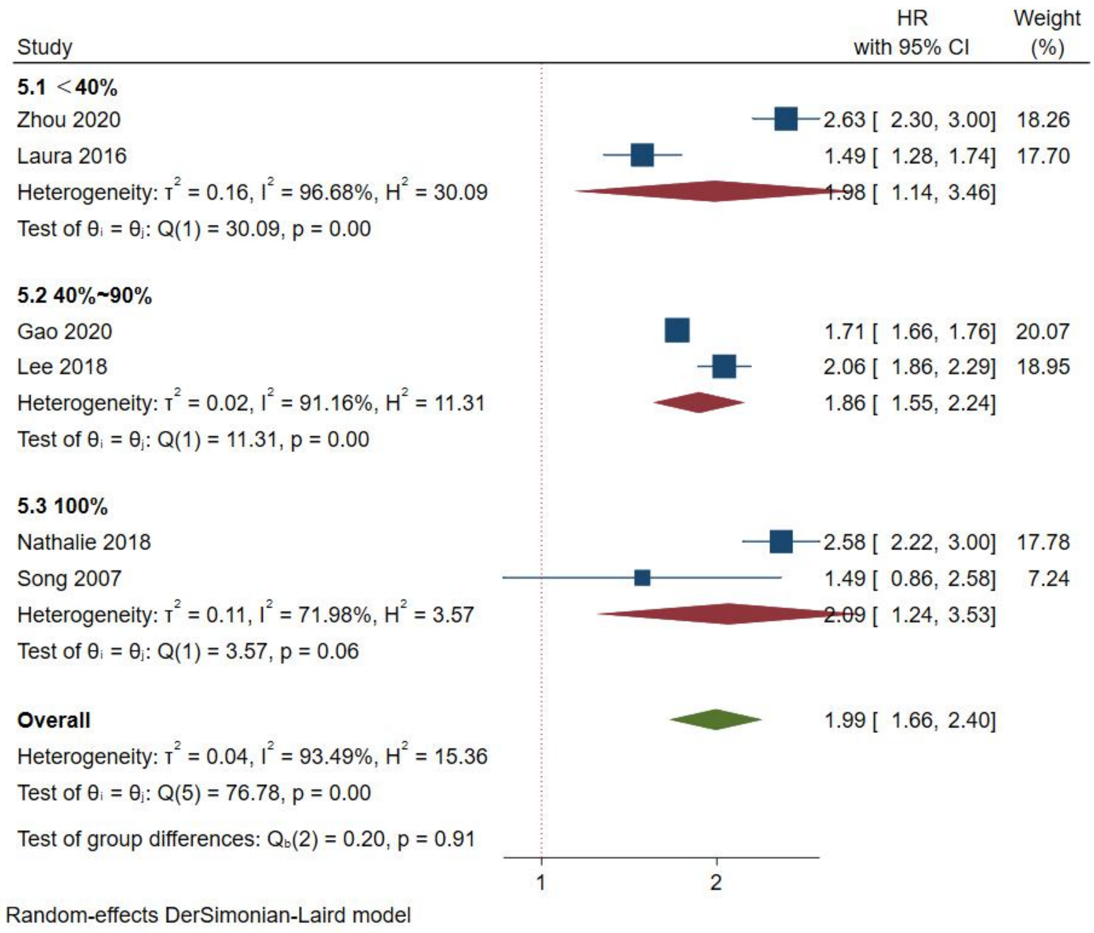

Figure S25 Subgroup (proportion of female) analysis of risk of stroke in MUO phenotypes compared with MHNW phenotypes

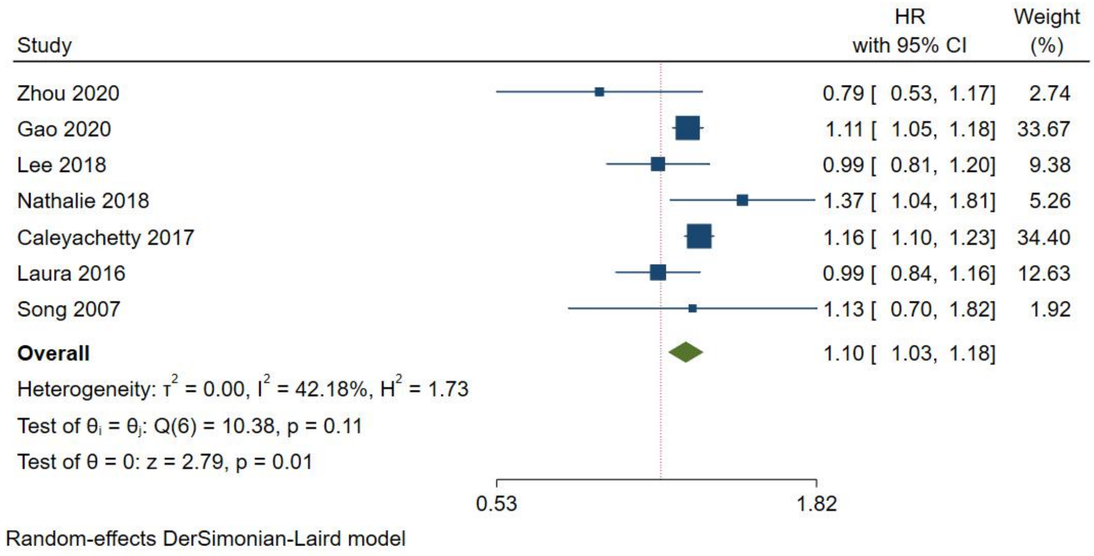

Figure S26 Results of sensitivity analysis in in terms of methodological quality

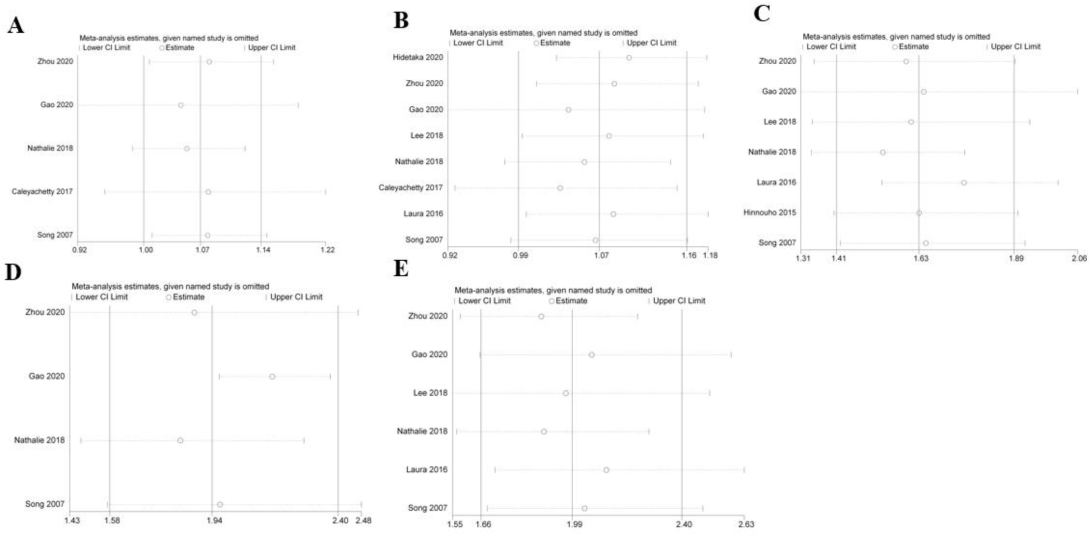

Figure S27. Results of sensitivity analysis by leave-one-out method
