# Supplementary material for: Risk of Stroke Among Different Metabolic Obesity Phenotypes: A Systematic Review and Meta-Analysis
Source: Front Cardiovasc Med. 2022 Apr 25;9:844550. doi: 10.3389/fcvm.2022.844550 (PMC9081493; doi:10.3389/fcvm.2022.844550)
Supplement: Supplementary file 3 [file Table_1.docx]

**Table S1.** Search strategy

| **PubMed** | |
| --- | --- |
| #1 | "cerebrovascular disorders"[MeSH Terms] |
| #2 | "stroke*"[Title/Abstract] OR "poststroke"[Title/Abstract] OR "apoplex*"[Title/Abstract] OR "cerebral vasc*"[Title/Abstract] OR "brain vasc*"[Title/Abstract] OR "cerebrovasc*"[Title/Abstract] OR "cva"[Title/Abstract] OR "SAH"[Title/Abstract] |
| #3 | ("brain"[Title/Abstract] OR "cerebr*"[Title/Abstract] OR "cerebell*"[Title/Abstract] OR "vertebrobasil*"[Title/Abstract] OR "hemispher*"[Title/Abstract] OR "intracran*"[Title/Abstract] OR "intracerebral"[Title/Abstract] OR "infratentorial"[Title/Abstract] OR "supratentorial"[Title/Abstract] OR "middle cerebral artery"[Title/Abstract] OR "mca"[Title/Abstract] OR "anterior circulation"[Title/Abstract] OR "posterior circulation"[Title/Abstract] OR "basilar artery"[Title/Abstract] OR "vertebral artery"[Title/Abstract] OR "space-occupying"[Title/Abstract]) AND ("ischaemi*"[Title/Abstract] OR "ischemi*"[Title/Abstract] OR "infarct*"[Title/Abstract] OR "thrombo*"[Title/Abstract] OR "emboli*"[Title/Abstract] OR "occlus*"[Title/Abstract] OR "hypoxi*"[Title/Abstract]) |
| #4 | ("brain*"[Title/Abstract] OR "cerebr*"[Title/Abstract] OR "cerebell*"[Title/Abstract] OR "intracerebral"[Title/Abstract] OR "intracran*"[Title/Abstract] OR "parenchymal"[Title/Abstract] OR "intraparenchymal"[Title/Abstract] OR "intraventricular"[Title/Abstract] OR "infratentorial"[Title/Abstract] OR "supratentorial"[Title/Abstract] OR "basal gangli*"[Title/Abstract] OR "putaminal"[Title/Abstract] OR "putamen"[Title/Abstract] OR "posterior fossa"[Title/Abstract] OR "hemispher*"[Title/Abstract] OR "subarachnoid"[Title/Abstract]) AND ("haemorrhag*"[Title/Abstract] OR "haemorrhag*"[Title/Abstract] OR "haematoma*"[Title/Abstract] OR "hematoma*"[Title/Abstract] OR "bleed*"[Title/Abstract]) |
| #5 | #1 or #2 or #3 or #4 |
| #6 | "obesity"[MeSH Terms] OR "obesity, morbid"[MeSH Terms] OR "obesity, abdominal"[MeSH Terms] OR "overweight"[MeSH Terms] |
| #7 | "adipos*"[Title/Abstract] OR "obes*"[Title/Abstract] OR "overweight*"[Title/Abstract] OR "over weight*"[Title/Abstract] OR "body mass ind*"[Title/Abstract] OR "BMI"[Title/Abstract] OR "waist hip ratio"[Title/Abstract] OR "skinfold thickness"[Title/Abstract] |
| #8 | #6 or #7 |
| #9 | "normal"[Title/Abstract] OR "healthy"[Title/Abstract] OR "benign"[Title/Abstract] OR "without"[Title/Abstract] OR "absence"[Title/Abstract] |
| #10 | metabolic*[Title/Abstract] |
| #11 | #8 and #9 and #10 |
| #12 | "obesity, metabolically benign"[MeSH Terms] |
| #13 | "obesity phenotype*"[Title/Abstract] OR "MHO"[Title/Abstract] |
| #14 | #11 or #12 or #33 |
| #15 | #5 and #14 |
| **Cochrane Library** | |
| #1 | MeSH descriptor: [Cerebrovascular Disorders] explode all trees |
| #2 | (stroke* or poststroke or apoplex* or cerebral vasc* or brain vasc* or cerebrovasc* or cva* or SAH):ti,ab,kw (Word variations have been searched) |
| #3 | (brain or cerebr* or cerebell* or vertebrobasil* or hemispher* or intracran* or intracerebral or infratentorial or supratentorial or middle cerebral artery or MCA* or anterior circulation or posterior circulation or basilar artery or vertebral artery or space‐occupying):ti,ab,kw (Word variations have been searched) |
| #4 | (ischaemi* or ischemi* or infarct* or thrombo* or emboli* or occlus* or hypoxi*):ti,ab,kw (Word variations have been searched) |
| #5 | #3 AND #4 |
| #6 | (brain* or cerebr* or cerebell* or intracerebral or intracran* or parenchymal or intraparenchymal or intraventricular or infratentorial or supratentorial or basal gangli* or putaminal or putamen or posterior fossa or hemispher* or subarachnoid):ti,ab,kw (Word variations have been searched) |
| #7 | (haemorrhag* or haemorrhag* or haematoma* or hematoma* or bleed*):ti,ab,kw (Word variations have been searched) |
| #8 | #6 AND #7 |
| #9 | #1 OR #2 OR #5 OR #8 |
| #10 | MeSH descriptor: [Obesity] explode all trees |
| #11 | MeSH descriptor: [Obesity, Morbid] explode all trees |
| #12 | MeSH descriptor: [Obesity, Abdominal] explode all trees |
| #13 | MeSH descriptor: [Overweight] explode all trees |
| #14 | (adipos* or obes* or overweight* or over weight* or body mass ind* or BMI or waist hip ratio or skinfold thickness):ti,ab,kw (Word variations have been searched) |
| #15 | #10 OR #11 OR #12 OR #13 OR #14 |
| #16 | (normal or healthy or benign or without or absence):ti,ab,kw (Word variations have been searched) |
| #17 | (metabolic*):ti,ab,kw (Word variations have been searched) |
| #18 | #15 AND #16 AND #17 |
| #19 | MeSH descriptor: [Obesity, Metabolically Benign] explode all trees |
| #20 | (obesity phenotype* or MHO):ti,ab,kw (Word variations have been searched) |
| #21 | #18 OR #19 OR #20 |
| #22 | #9 AND #21 |
| **Embase** | |
| #1 | 'cerebrovascular disease'/exp |
| #2 | stroke*:ab,ti OR poststroke:ab,ti OR apoplex*:ab,ti OR 'cerebral vasc*':ab,ti OR 'brain vasc*':ab,ti OR cerebrovasc*:ab,ti OR cva*:ab,ti OR sah:ab,ti |
| #3 | brain:ab,ti OR cerebr*:ab,ti OR cerebell*:ab,ti OR vertebrobasil*:ab,ti OR hemispher*:ab,ti OR intracran*:ab,ti OR intracerebral:ab,ti OR infratentorial:ab,ti OR supratentorial:ab,ti OR 'middle cerebral artery':ab,ti OR mca*:ab,ti OR 'anterior circulation':ab,ti OR 'posterior circulation':ab,ti OR 'basilar artery':ab,ti OR 'vertebral artery':ab,ti OR space‐occupying:ab,ti |
| #4 | ischaemi*:ab,ti OR ischemi*:ab,ti OR infarct*:ab,ti OR thrombo*:ab,ti OR emboli*:ab,ti OR occlus*:ab,ti OR hypoxi*:ab,ti |
| #5 | #3 AND #4 |
| #6 | brain:ab,ti OR cerebr*:ab,ti OR cerebell*:ab,ti OR parenchymal:ab,ti OR intraparenchymal:ab,ti OR intracran*:ab,ti OR intracerebral:ab,ti OR infratentorial:ab,ti OR intraventricular:ab,ti OR supratentorial:ab,ti OR 'basal gangli*':ab,ti OR putaminal:ab,ti OR putamen:ab,ti OR 'posterior fossa':ab,ti OR hemispher*:ab,ti OR subarachnoid:ab,ti |
| #7 | haemorrhag*:ab,ti OR haematoma*:ab,ti OR hematoma*:ab,ti OR bleed*:ab,ti |
| #8 | #6 AND #7 |
| #9 | #1 OR #2 OR #5 OR #8 |
| #10 | 'obesity'/exp OR 'morbid obesity'/exp OR 'abdominal obesity'/exp |
| #11 | adipos*:ab,ti OR obes*:ab,ti OR overweight*:ab,ti OR 'over weight*':ab,ti OR 'body mass ind*':ab,ti OR bmi:ab,ti OR 'waist hip ratio':ab,ti OR 'skinfold thickness':ab,ti |
| #12 | #10 OR #11 |
| #13 | normal:ab,ti OR healthy:ab,ti OR benign:ab,ti OR without:ab,ti OR absence:ab,ti |
| #14 | metabolic*:ab,ti |
| #15 | #12 AND #13 AND #14 |
| #16 | 'metabolically benign obesity'/exp |
| #17 | 'obesity phenotype*':ab,ti OR mho:ab,ti |
| #18 | #15 OR #16 OR #17 |
| #19 | #9 AND #18 |
