# Supplementary material for: Risk of Stroke Among Different Metabolic Obesity Phenotypes: A Systematic Review and Meta-Analysis
Source: Front Cardiovasc Med. 2022 Apr 25;9:844550. doi: 10.3389/fcvm.2022.844550 (PMC9081493; doi:10.3389/fcvm.2022.844550)
Supplement: Supplementary file 4 [file Table_2.docx]

**Table S2. The full statistics of meta-analyses**

| **Study** | **MHOW** | | **MHNW** | | **HR (95% CI)** | ***I^2^*(%)** |
| --- | --- | --- | --- | --- | --- | --- |
|  | **Events** | **Total** | **Events** | **Total** |  |  |
| Zhou 2020 | NA | NA | 308 | 17367 | 0.96(0.78-1.18) | / |
| Gao 2020 | 8352 | 107517 | 17697 | 232975 | 1.10(1.08-1.13) | / |
| Nathalie 2018 | 153 | 15583 | 255 | 50336 | 1.29(1.05-1.58) | / |
| Caleyachetty 2017 | 10097 | 899471 | 10918 | 1318516 | 1.03(0.99-1.08) | / |
| Song 2007 | 56 | 6730 | 129 | 12943 | 0.83(0.58-1.18) | / |
| **Total** | 18658 | 1029301 | 29307 | 1632137 | 1.07(1.00-1.14) | 69.50 |
|  | **MHO** | | **MHNW** | | **HR (95% CI)** | ***I^2^*(%)** |
|  | **Events** | **Total** | **Events** | **Total** |  |  |
| Hidetaka 2020 | NA | NA | NA | NA | 0.86(0.68-1.08) | / |
| Zhou 2020 | NA | NA | 308 | 17367 | 0.79(0.53-1.17) | / |
| Gao 2020 | 1064 | 15044 | 17697 | 232975 | 1.11(1.05-1.18) | / |
| Lee 2018 | 121 | 26448 | 465 | 110531 | 0.99(0.81-1.20) | / |
| Nathalie 2018 | 60 | 5749 | 255 | 50336 | 1.37(1.04-1.81) | / |
| Caleyachetty 2017 | 7526 | 517444 | 10918 | 1318516 | 1.16(1.10-1.23) | / |
| Laura 2016 | 18 | 642 | 88 | 3243 | 0.99(0.84-1.16) | / |
| Song 2007 | 22 | 2925 | 129 | 12943 | 1.13(0.70-1.82) | / |
| **Total** | 8811 | 568252 | 29860 | 1745911 | 1.07(0.99-1.16) | 54.82 |
|  | **MUNW** | | **MHNW** | | **HR (95% CI)** | ***I^2^*(%)** |
|  | **Events** | **Total** | **Events** | **Total** |  |  |
| Zhou 2020 | 1310 | 27073 | 308 | 17367 | 1.80(1.58-2.05) | / |
| Gao 2020 | 2905 | 18428 | 17697 | 232975 | 1.54(1.48-1.60) | / |
| Lee 2018 | 2535 | 130583 | 465 | 110531 | 1.72(1.55-1.90) | / |
| Nathalie 2018 | 1071 | 8629 | 255 | 50336 | 2.22(1.92-2.57) | / |
| Laura 2016 | 33 | 805 | 88 | 3243 | 1.20(1.09-1.33) | / |
| Hinnouho 2015 | 16 | 649 | 42 | 3100 | 1.62(0.89-2.94) | / |
| Song 2007 | 12 | 583 | 129 | 12943 | 1.24(0.64-2.40) | / |
| **Total** | 7882 | 186750 | 18984 | 430495 | 1.63(1.41-1.89) | 89.74 |
|  | **MUOW** | | **MHNW** | | **HR (95% CI)** | ***I^2^*(%)** |
|  | **Events** | **Total** | **Events** | **Total** |  |  |
| Zhou 2020 | NA | NA | 308 | 17367 | 2.11(1.87-2.39) | / |
| Gao 2020 | 7815 | 50158 | 17697 | 232975 | 1.64(1.61-1.68) | / |
| Nathalie 2018 | 899 | 5708 | 255 | 50336 | 2.27(1.96-2.62) | / |
| Song 2007 | 19 | 1104 | 129 | 12943 | 1.74(1.05-2.88) | / |
| **Total** | 8733 | 56970 | 18389 | 313621 | 1.94(1.58-2.40) | 91.17 |
|  | **MUO** | | **MHNW** | | **HR (95% CI)** | ***I^2^*(%)** |
|  | **Events** | **Total** | **Events** | **Total** |  |  |
| Zhou 2020 | NA | NA | 308 | 17367 | 2.63(2.30-3.00) | / |
| Gao 2020 | 5118 | 34124 | 17697 | 232975 | 1.71(1.66-1.76) | / |
| Lee 2018 | 1763 | 86521 | 465 | 110531 | 2.06(1.86-2.29) | / |
| Nathalie 2018 | 642 | 4252 | 255 | 50336 | 2.58(2.22-3.00) | / |
| Laura 2016 | 23 | 481 | 88 | 3243 | 1.49(1.28-1.74) | / |
| Song 2007 | 18 | 1341 | 129 | 12943 | 1.49(0.86-2.58) | / |
| **Total** | 7564 | 126719 | 18942 | 427395 | 1.99(1.66-2.40) | 93.49 |

(NA: Not reported)
